# Mendelian randomization highlights sleep disturbances mediated the effect of depression on chronic pain

**DOI:** 10.1002/brb3.3596

**Published:** 2024-07-05

**Authors:** Yingchao Zhu, Yaodan Bi, Tao Zhu

**Affiliations:** ^1^ Department of Anesthesiology, West China Hospital Sichuan University Chengdu Sichuan China; ^2^ Department of Anesthesiology, Peking Union Medical College Hospital Peking Union Medical College and Chinese Academy of Medical Sciences Beijing China

**Keywords:** chronic pain, depression, Mendelian randomization (MR), sleep disturbances

## Abstract

**Introduction:**

Depression and chronic pain are significant contributors to the global burden of disease. Previous research has revealed complex relationships between these two conditions, which may be influenced by sleep quality. However, observational studies have limitations, including confounding factors and reverse causation. This study aims to explore the mediating effects of sleep on the relationship between depression and chronic pain using Mendelian randomization (MR).

**Methods:**

We conducted a two‐step, two‐sample MR study using mediation analysis. We obtained major depressive disorder (MDD) Genome‐Wide Association Studdies (GWAS) data from Wray et al.’s GWAS meta‐analysis. Phenotypic data related to sleep were collected from the UK Biobank. Chronic pain data were obtained from the Finnish database.

**Results:**

MR analysis revealed significant genetic associations between MDD and chronic localized pain [IVW: odds ratio (OR) = 1.26, 95% confidence interval (CI) = 1.16–1.38, *p* = 2.52 × 10^–7^] as well as fibromyalgia (IVW: OR = 2.17, 95% CI = 1.34–3.52, *p* = .002). Genetic susceptibility for MDD was also associated with insomnia (IVW: OR = 1.10, 95% CI = 1.06–1.13, *p* = 3.57 × 10^–8^) and self‐reported short sleep duration (IVW: OR = 1.03, 95% CI = 1.00–1.06, *p* = .047). The mediating effects of insomnia and fibromyalgia on the pathway from depression to chronic regional pain were 1.04 and 1.03, respectively, with mediation proportions of 12.8% and 15.2%. Insomnia mediated the pathway between depression and fibromyalgia with an effect of 1.12, accounting for 15.2% of the total effect.

**Conclusion:**

This two‐step MR analysis strengthens the evidence of genetic predictive associations between depression and chronic pain, highlighting the mediating roles of insomnia and short sleep duration. It further elucidates the specific roles of distinct sleep disorders, differentiating insomnia and short sleep duration from other sleep‐related phenotypes.

## Graphical abstract

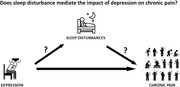

## 1 INTRODUCTION

Depression and pain play important roles in the global disease burden (GBD 2017 Disease & Injury Incidence & Prevalence Collaborators, 2018). A study conducted in European countries found that nearly 30% of individuals with severe depression reported experiencing pain attacks (Ohayon & Schatzberg, 2003). Long‐term research related to the impact of depression on pain indicates a significant reduction in pain after depressive symptoms are alleviated (Gerrits et al., 2015; Mossey & Gallagher, 2004; Skadberg et al., 2020).

However, the relationship between depression and pain may be influenced by other factors, such as sleep quality. Sleep disorders often cooccur with depression (Murphy & Peterson, 2015). Previous studies (Afolalu et al., 2018; Koffel et al., 2016; Stocks et al., 2018) have demonstrated a significant correlation between sleep quality and pain. Specifically, individuals who suffer from sleep disorders have a 50% greater likelihood of experiencing pain. These findings suggest a possible interrelationship between depression, pain, and sleep disorders. A cross‐sectional analysis targeting adolescents found a close correlation between short sleep duration, depressive symptoms, and pain. A study on patients with multiple sclerosis revealed that the impact of chronic pain on depression was largely mediated by fatigue, anxiety, and sleep disorders. Although these observational studies (Amtmann et al., 2015; Haraldstad & Stea, 2021; Ravyts et al., 2019) explored different research directions and sample populations, they all confirmed the association among the three factors. A meta‐analysis conducted by Karimi et al. (2023) demonstrated that sleep disorders played a mediating role of approximately 12.5% between depression and chronic pain. However, both observational studies and their meta‐analyses may be influenced by potential confounding factors and reverse causation.

Given the simultaneous presence of pain, sleep, and depression, they collectively have a significant adverse impact on individuals and society. To elucidate the interrelationships among these three factors, the Mendelian randomization (MR) method can provide more reliable results. The MR method yields robust results that are closer to those obtained from randomized controlled trials and are less susceptible to confounding bias, measurement errors, and reverse causality. Previous MR analyses have shown a significant association between depression and widespread chronic pain as well as multisite pain (Tang et al., 2022; Zhao et al., 2023). There have also been MR studies exploring the relationship between depression and insomnia (Sun et al., 2022), but the relationship between depression and other sleep‐related phenotypes has not been extensively studied. Additionally, previous MR analyses only investigated the correlation between insomnia, daytime sleepiness, and low back pain (Shu et al., 2022), while the relationship between other sleep‐related phenotypes and pain in other body regions or widespread chronic pain still needs further research. In recent times, the MR method has been employed to investigate mediating pathways. Compared to traditional multivariable approaches, the two‐step method has higher sensitivity in evaluating potential mediating factors and is less likely to induce inherent biases.

The aim of this study was to apply the MR framework to investigate the effects of depression and sleep disturbances on risk of chronic pain. For sleep disturbances risk factors that the MR analyses supported to have a causal effect on chronic pain risk, we aimed to further apply MR mediation analyses to investigate the degree to which these factors might be mediating the effects of chronic pain attainment.

## 2 MATERIAL AND METHODS

### 2.1 Study design

We conducted a two‐step, two‐sample Mendelian randomization study using mediation analysis. The first step of our two‐step MR study is to investigate the association of genetically predicted major depressive disorder (MDD) with each genetically determined sleep mediator. In the second step, the approach investigated the association of these genetically determined sleep mediators with genetically predicted chronic pain risk after adjusting for MDD. The proportion of mediation was also estimated for each sleep mediator following the second step. Our study was conducted in accordance with the Declaration of Helsinki revised in 2013, and the methods followed the STROBE‐MR checklist (Skrivankova et al., 2021; Skrivankova et al., 2021). This study does not involve human subject research. Since our MR study is based on publicly available aggregated statistical data, ethical approval or informed consent is not required.

### 2.2 Data sources

We drew on summary statistics from the largest and most recent Genome‐Wide Association Studdies (GWAS) for MDD (*n* = 480,359) (Wray et al., 2018), defined as major depression based primarily on structured assessments by structured diagnostic interviews, electronic medical records, or self‐reported diagnosis or treatment for clinical depression by a medical professional. GWAS of sleep‐related phenotypes were based on data from the UK Biobank, a population‐based prospective study. We examined various sleep‐related phenotypes, including self‐reported traits such as insomnia (Lane et al., 2019), chronotype (Jones et al., 2019), morning person (Jones et al., 2019), sleep duration (Dashti et al., 2019), and short sleep duration (Dashti et al., 2019), as well as estimates derived from accelerometer data (Jones et al., 2019) such as sleep duration, number of sleep episodes, and least active 5 h (L5) timing (see Supplementary Tables for detailed data sources).

The summary statistics for chronic pain were obtained from FinnGen (https://storage.googleapis.com/finngen‐public‐data‐r8). The chronic pain dataset included information on two specific conditions: chronic regional pain and fibromyalgia. The summary statistics for chronic regional pain included 341,797 participants, with cases defined as individuals carrying ICD‐10 diagnosis codes for disorders with substantial pain symptoms, such as pain in the limbs, back, neck, head, and abdomen. The fibromyalgia summary statistics included 255,063 participants and encompassed diseases of the musculoskeletal system and connective tissue. All GWASs were adjusted for sex, age, and study‐specific variables.

### 2.3 Selection and validation of SNPs

SNPs that meet a significance threshold of *p* < 5 × 10^–8^ were selected as instrumental variables (IVs). To ensure variable independence and account for linkage disequilibrium (LD) effects, an LD parameter (*r* ^2^) of 0.001 and a genetic distance of 10,000 kb were utilized. We removed palindromic SNPs from the instrumental single nucleotide polymorphisms single nucleoti(SNPs) that were chosen for analysis. The F statistic was employed to exclude weak instrumental biases, with SNPs having an F statistic < 10 being excluded (Pierce et al., 2011).

### 2.4 MR analysis

The primary analysis method employed was the inverse variance‐weighted (IVW) approach (Burgess et al., 2013). Additionally, MR‐Egger, weighted median, simple mode, and weighted mode methods were utilized. The estimates were reported as odds ratios (ORs) along with their corresponding 95% confidence intervals (CIs). Bonferroni correction was applied as the threshold of significance for correcting the multiple testing. A nominal significance level of *p* < .05 was also considered. The reverse MR analysis procedure was conducted following the same steps as mentioned above.

### 2.5 Pleiotropy and heterogeneity analysis

Cochran's *Q* test was performed to assess heterogeneity among individual causal effects, with significance defined as a Q_*p*‐value < .05, or *I* ^2^ statistics > 25% indicating heterogeneity. We utilized MR‐Egger regression to evaluate whether there is a presence of directional pleiotropy of instrumental variables (Bowden et al., 2015). We performed Mendelian randomization pleiotropy residual sum and outlier (MR‐PRESSO) analysis and leave‐one‐out analysis to identify any outlier instrumental variables (Verbanck et al., 2018). We performed stepwise removal of instrumental variables identified as outliers by MR‐PRESSO to mitigate the impact of horizontal pleiotropy. If one or more outlier SNPS were found to affect the MR Estimates, they were removed and MR analysis was performed again.

## 3 RESULTS

**FIGURE 1:**
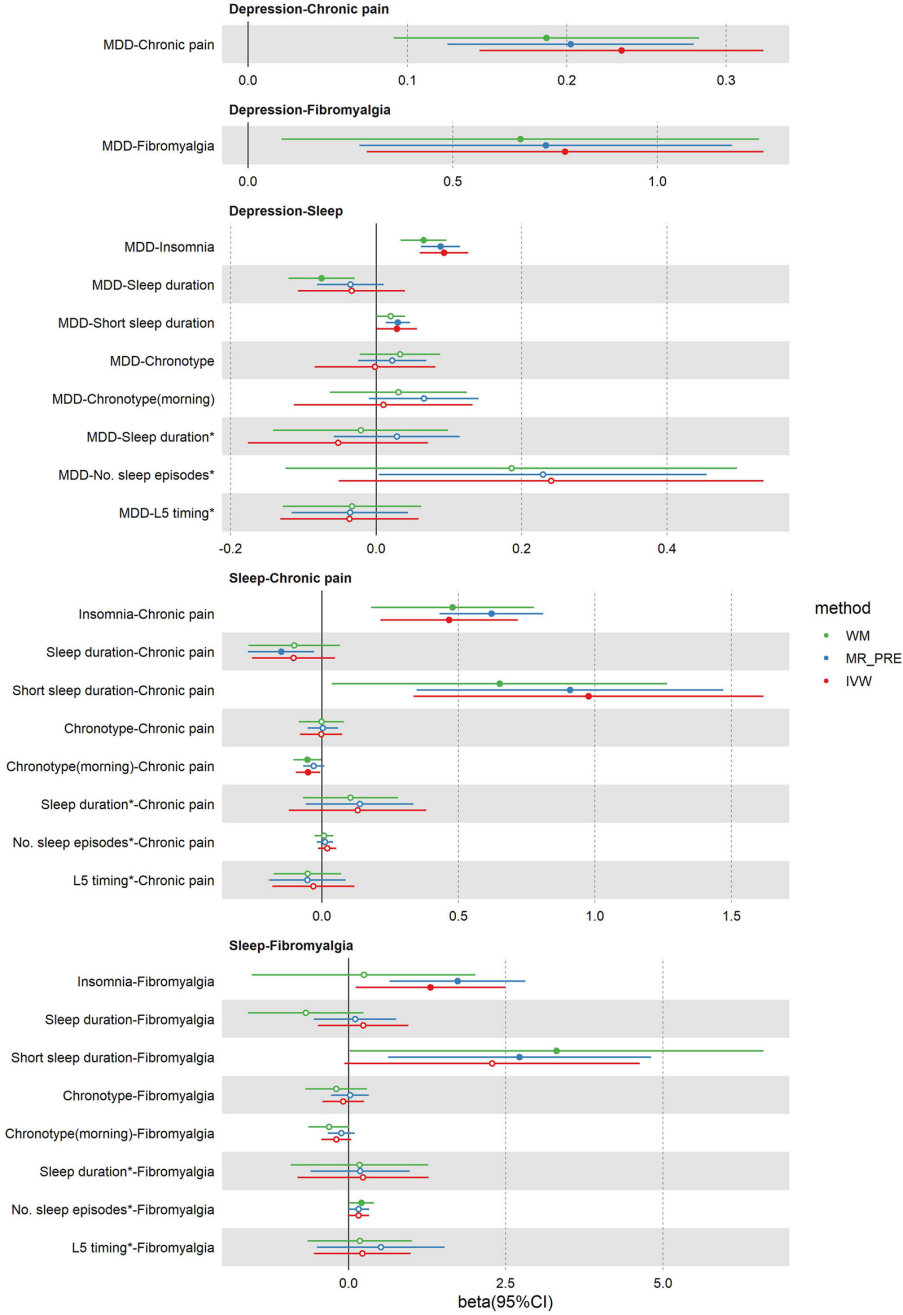
Estimate of the effect of genetically predicted MDD on genetically predicted sleep subtypes and chronic pain, as well as the genetically predicted sleep subtypes on chronic pain. Note: MDD, major depressive disorder; WM, weighted median; MR_PRE, When the MR‐PRESSO test detects outliers, it reports the value of “adjusted MR‐PRESSO.” In the absence of outliers, it reports the value of “raw MR‐PRESSO method”; IVW, inverse variance weighted; L5 timing, least active 5 h timing; No. sleep episodes, the number of nocturnal sleep episodes; *, accelerometer‐based sleep‐related phenotypes.

The harmonies data and full results of the MR analysis are listed in the Supplementary Tables. With the utilization of MR analysis (Figure 1), MDD was associated with a higher risk of chronic regional pain [IVW: odds ratio (OR) = 1.26, 95% confidence interval (CI) = 1.16–1.38, *p* = 2.52 × 10^–7^] and fibromyalgia (IVW: OR = 2.17, 95% CI = 1.34–3.52, *p* = .002). Genetic liability to MDD also increased the odds of two sleep‐related phenotypes, including insomnia (IVW: OR = 1.10, 95% CI = 1.06–1.13, *p* = 3.57 × 10^–8^) and self‐reported short sleep duration (IVW: OR = 1.03, 95% CI = 1.00–1.06, *p* = .047). Since there was no evidence of association of MDD was associated with other genetically predicted sleep‐related phenotypes, including self‐reported chronotype, morning person, sleep duration, accelerometer‐based sleep duration, number of sleep episodes, and L5 timing risk in the IVW MR analysis (*p* > .05), these potential mediators were excluded. Mediation effect of insomnia on MDD and both two chronic pain traits were prominent, while mediation effect of self‐reported short sleep duration on MDD and fibromyalgia were not significant (*p* > .05). The mediation effect of insomnia and fibromyalgia in the pathway from MDD to chronic regional pain was 1.04 and 1.03, with a mediation proportion of 12.8% and 15.2%, respectively. The mediation effect of insomnia in the causal pathway from MDD to fibromyalgia was 1.12 accounting for 15.2% of the total effect. Results of adjusted MR‐PRESSO (*p* < .05) indicated that our results were less likely biased by invalid instruments. All Egger regression results indicated there were not horizontal pleiotropies existing (*p* < .05 with nonzero Egger intercept).

## 4 DISCUSSION

In this study, we used Mendelian randomization to investigate the relationship between depression, sleep disturbances, and chronic pain, and further explored the mediating role of sleep disturbances. Our results support a significant association between depression and chronic pain. Through MR analysis, we found a positive relationship between depression and chronic regional pain as well as fibromyalgia, with IVW ORs of 1.26 and 2.17, respectively. This finding is consistent with previous MR studies (Tang et al., 2022; Zhao et al., 2023), which have also reported significant associations between depression and widespread chronic pain and multisite pain. This suggests that depression may be an important risk factor for chronic pain. Our study also identified associations between depression and two sleep‐related phenotypes, namely, insomnia and self‐reported short sleep duration. This association may partially explain the relationship between depression and chronic pain. However, other sleep disturbances phenotypes (including self‐reported chronotype, morning person, sleep duration, accelerometer‐based sleep duration, number of sleep episodes, and L5 timing risk) did not mediate the relationship between depression and chronic pain. These results highlight the critical role of insomnia and short sleep duration in the process of depression leading to chronic pain and provide new insights into understanding this complex relationship.

Previous studies have identified the association between depression, sleep disturbances, and chronic pain, but these studies are often influenced by potential confounders and reversed causality. In contrast, our study for the first time employed the MR method to analyze the mediating effect of sleep disturbances, reducing the potential bias, measurement errors, and reversed causality, thus providing more reliable results. Moreover, compared to previous studies, our research further refined the types of sleep disturbances, affirming the mediating roles of insomnia while differentiating them from other types of sleep disturbances. This provides more specific information for public health policymakers and healthcare providers to develop more effective strategies and reduce the incidence of depression‐related chronic pain. Choosing phenotype‐based SNPs as genetic instruments has the advantages of easy accessibility and high biological relevance, as they can be obtained from large‐scale population samples through methods such as questionnaires, clinical assessments, or biomarkers, and are typically associated with the biological mechanisms of diseases or symptoms. However, this choice also carries the risk of subjectivity and measurement bias, as phenotype‐based SNPs may be influenced by patient‐reported symptoms or clinical assessments, thereby increasing the risk of inconsistency and error in the results.

Certain mechanisms may explain how sleep disturbances mediate the impact of depression on chronic pain. Neurobiological pathways are pivotal in this process, as depression alters neurotransmitter systems like serotonin, norepinephrine, and dopamine, crucial for pain modulation (Finan & Smith, 2013; Léna et al., 2005; Meyers et al., 2011). Sleep disturbances, notably insomnia, exacerbate this by further disrupting neurotransmitter systems and impacting neuroplasticity and neuroinflammatory processes, fostering chronic pain development and maintenance (Meyers et al., 2011). Psychophysiological factors, including alterations in stress response systems and impaired cognitive functioning, also contribute to this mediation effect (Meyers et al., 2011). Moreover, immune dysregulation, characterized by systemic inflammation and altered cytokine profiles due to sleep disturbances, perpetuates chronic pain and depressive symptoms (Haack et al., 2007; Irwin & Miller, 2007; Irwin et al., 2010). Central sensitization mechanisms (Hubbard et al., 2014; May, 2008; Woolf, 2011), involving heightened central nervous system excitability and alterations in pain‐processing regions, further amplify pain perception in individuals with depression experiencing sleep disturbances. These interconnected pathways illustrate the complex interplay between sleep disturbances, depression, and chronic pain, highlighting the need for multifaceted interventions targeting these mechanisms. Expanding on these findings, a bidirectional relationship between sleep disturbances and depression is evident. Chronic pain disrupts sleep architecture, leading to fragmented sleep patterns, reduced sleep efficiency, and increased sleep latency (Finan et al., 2013; Smith & Haythornthwaite, 2004). Conversely, inadequate or poor‐quality sleep exacerbates depressive symptoms, further contributing to chronic pain development and maintenance. This bidirectional relationship emphasizes the importance of addressing sleep disturbances as a vital component of comprehensive treatment approaches for individuals with depression and chronic pain. Individual differences in vulnerability to sleep disturbances, depression, and chronic pain necessitate personalized intervention approaches. Genetic predisposition (Schneider et al., 2019), early life experiences, psychosocial stressors (McEwen & Gianaros, 2010), and comorbid medical conditions (Broen et al., 2012) can influence susceptibility to sleep disturbances and the development of depression and chronic pain. Tailoring interventions to target specific vulnerabilities and addressing underlying contributing factors can optimize treatment outcomes and improve overall well‐being in affected individuals. Interdisciplinary collaboration is essential, considering the complex interplay between biological, psychological, and social factors in sleep disturbances, depression, and chronic pain. Integrating medical, psychological, and behavioral interventions, including pharmacotherapy, cognitive‐behavioral therapy, mindfulness‐based interventions, and physical therapy, can provide comprehensive care to address the multifaceted nature of these conditions (Cojocaru et al., 2024; Kemani et al., 2024). By addressing underlying mechanisms and contributing factors through interdisciplinary approaches, healthcare providers can enhance treatment effectiveness and improve the quality of life for individuals affected by sleep disturbances, depression, and chronic pain.

Despite the use of the MR method, this study still has some limitations. First, while our MR study primarily focuses on the genetic determinants of MDD, sleep disturbances, and chronic pain, we acknowledge the potential influence of environmental risk factors. We address this issue by carefully selecting genetic instruments, conducting sensitivity analyses, and providing cautious interpretation of our results within the Mendelian randomization framework. Although MR has advantages in addressing confounding by environmental factors, we acknowledge the inability to fully capture the complex interactions between genetics and the environment. Therefore, we interpret our findings cautiously, recognizing that our study primarily elucidates the genetic pathways underlying the associations between MDD, sleep disturbances, and chronic pain, rather than directly assessing the effects of environmental risk factors. Second, it should be acknowledged that our study mainly focused on the European population, which may have different epidemiological and genetic characteristics compared to other populations, thus requiring cautious interpretation of the results. In addition, our study mainly focuses on sleep disorders as a mediating factor, but there may be other potential mediating factors that need further investigation, such as fatigue and anxiety. Future research needs to further evaluate the effectiveness of controlling these mediating factors in reducing the risk of chronic pain and gain a more comprehensive understanding of other related mediating factors and their interrelationships.

## 5 CONCLUSION

This two‐step MR analysis supports a genetically predicted association between depression and chronic pain, with insomnia and short sleep duration as a significant mediator. Our findings have the potential to inform strategies for mitigating the prevalence of chronic pain associated with depression and underscore the importance of understanding relevant mediating factors.

## AUTHOR CONTRIBUTIONS

**Yingchao Zhu**: Conceptualization; visualization; project administration; data curation; formal analysis; methodology; writing—original draft; writing—review and editing. **Yaodan Bi**: Visualization; project administration; data curation; formal analysis; methodology; writing—review and editing. **Tao Zhu**: project administration; data curation; formal analysis; methodology; writing—review and editing.

## CONFLICT OF INTEREST STATEMENT

The authors declare no conflicts of interest.

### PEER REVIEW

The peer review history for this article is available at https://publons.com/publon/10.1002/brb3.3596.

## FUNDING

This research received no specific grant from any funding agency in the public, commercial, or not‐for‐profit sectors.

## Supporting information

Supporting Information

Supporting Information

## Data Availability

The major depression data that support the findings of this study are openly available in the GWAS catalog at www.ebi.ac.uk/gwas, reference number: 29700475. The sleep‐related phenotypes data that support the findings of this study are openly available in the GWAS catalog at www.ebi.ac.uk/gwas, reference number: 30804566, 30952852, and 30846698. The chronic pain and fibromyalgia data that support the findings of this study are openly available in FinnGen database at https://r8.finngen.fi, reference name: PAIN and M13_FIBROMYALGIA.
