# Supplementary material for: Mendelian randomization highlights sleep disturbances mediated the effect of depression on chronic pain
Source: Brain Behav. 2024 Jul 5;14(7):e3596. doi: 10.1002/brb3.3596 (PMC11224770; doi:10.1002/brb3.3596)
Supplement: Supplementary file 1 — Supporting Information [file BRB3-14-e3596-s002.docx]

**Supplementary Table S1. Description of the sleep genome-wide association studies (GWAS) included in the two-sample MR analyses.**

MR, Mendelian randomization; SNP, single nucleotide polymorphism; No. sleep episodes, the number of nocturnal sleep episodes L5 timing, least active 5 h timing.

**Supplementary Table S2. MR-Egger regression analyses on detecting directional pleiotropy of Mendelian randomization.**

**Supplementary Table S3.Full result of MR analysis**.
