# Supplementary figures and images for: Mendelian randomization highlights sleep disturbances mediated the effect of depression on chronic pain
Source: Brain Behav. 2024 Jul 5;14(7):e3596. doi: 10.1002/brb3.3596 (PMC11224770; doi:10.1002/brb3.3596)

# MR Test

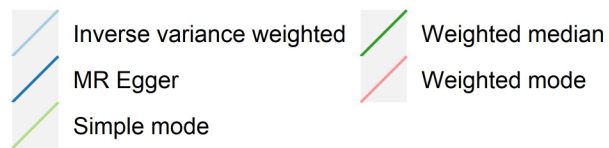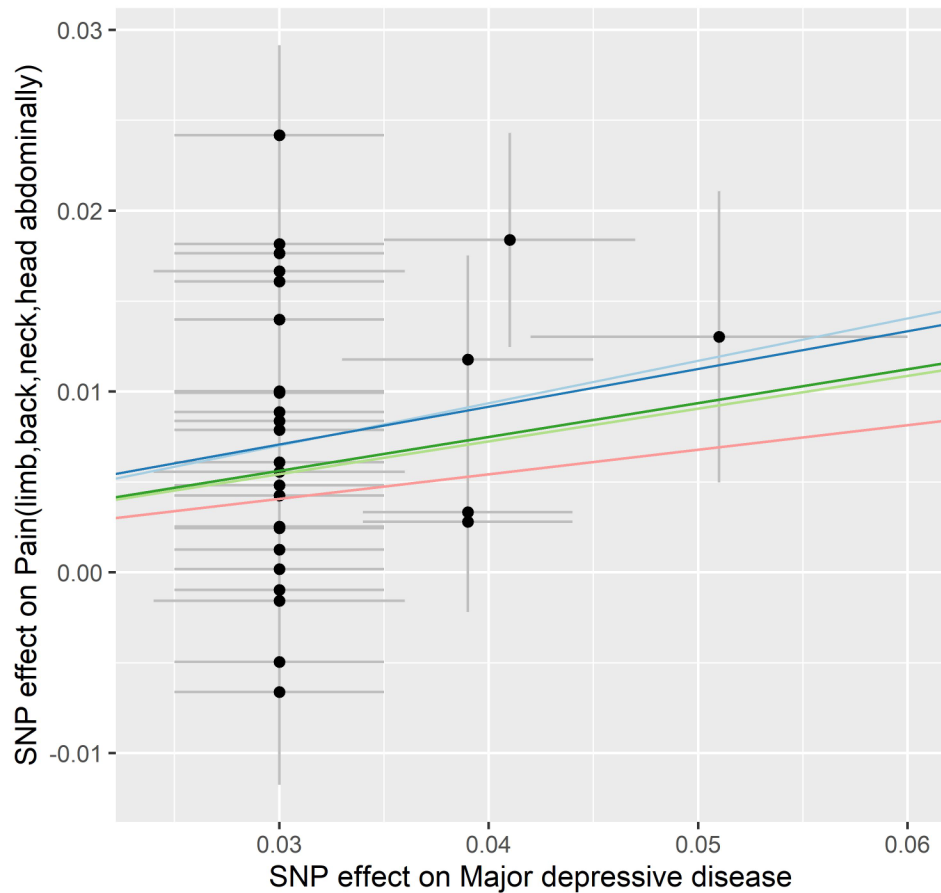

# MR Test

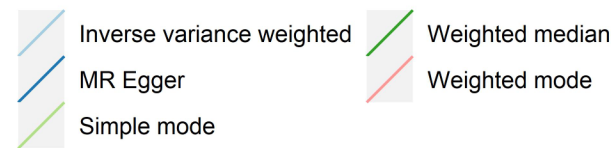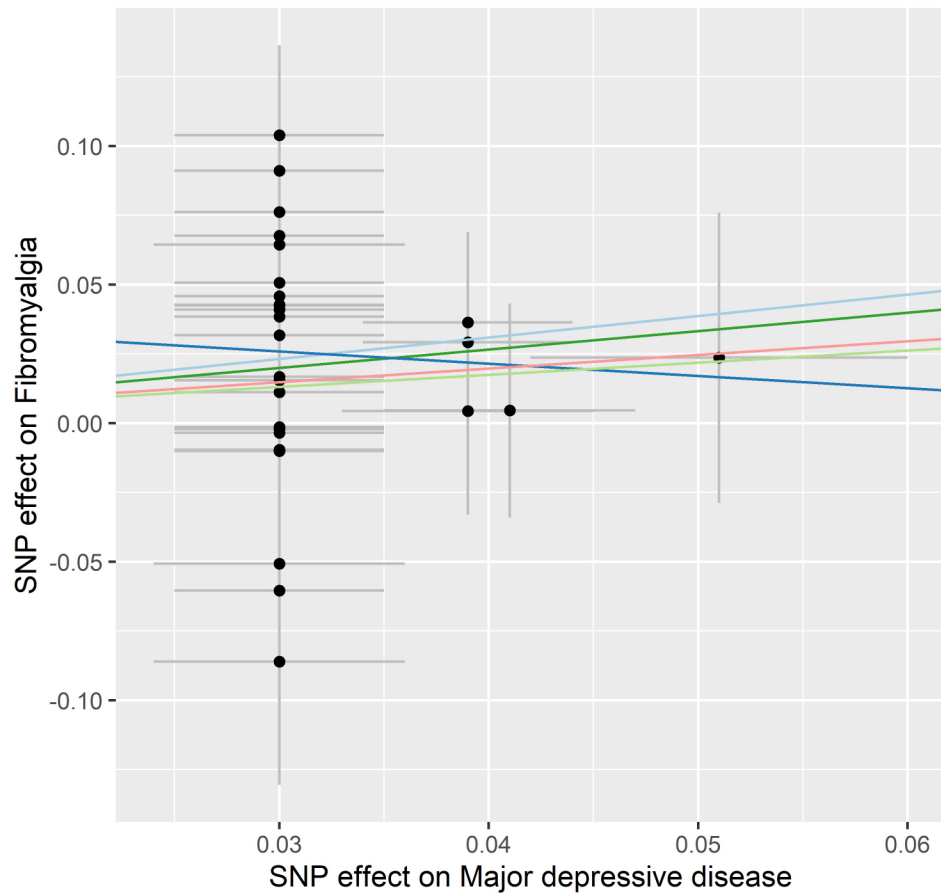

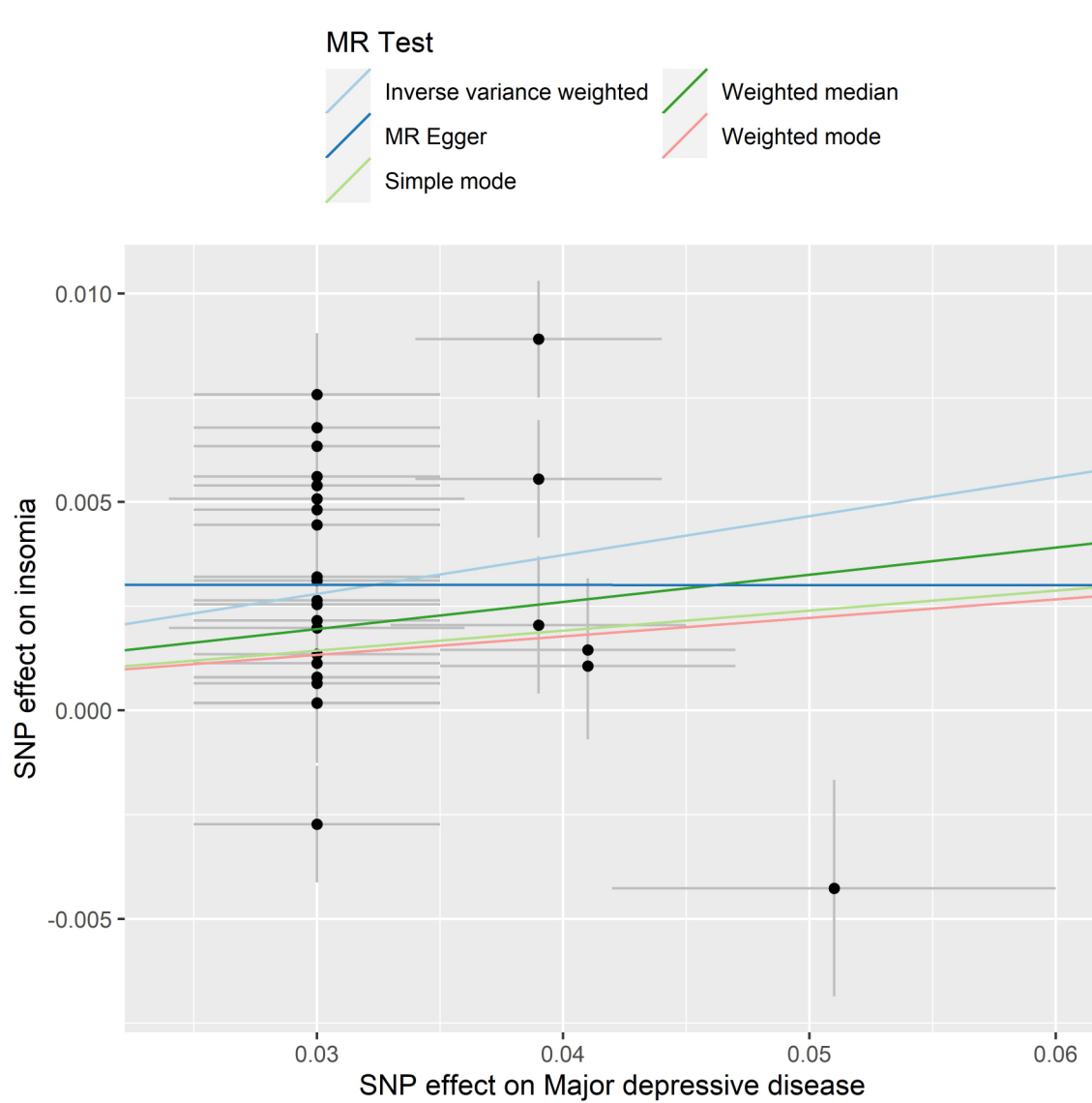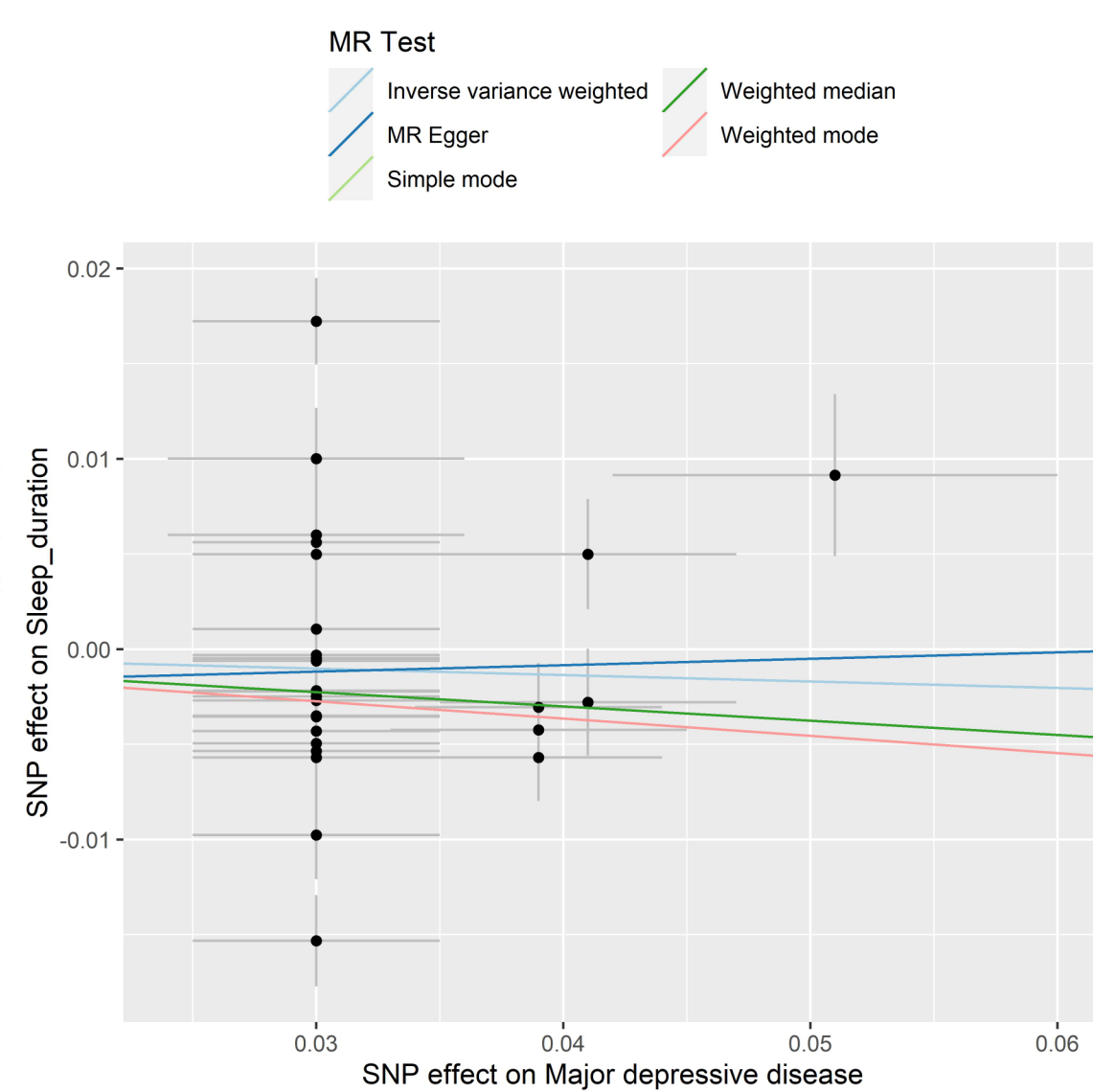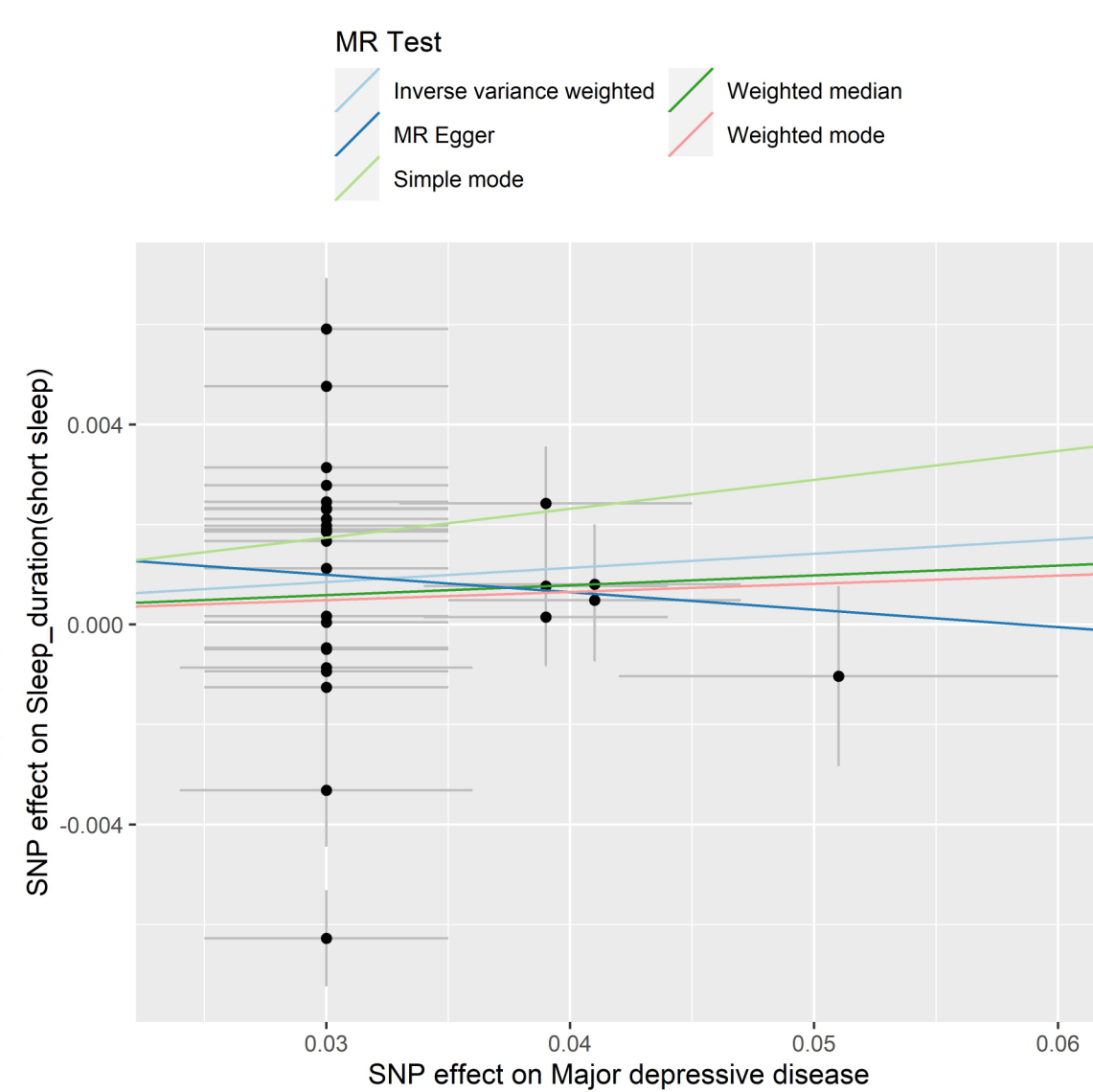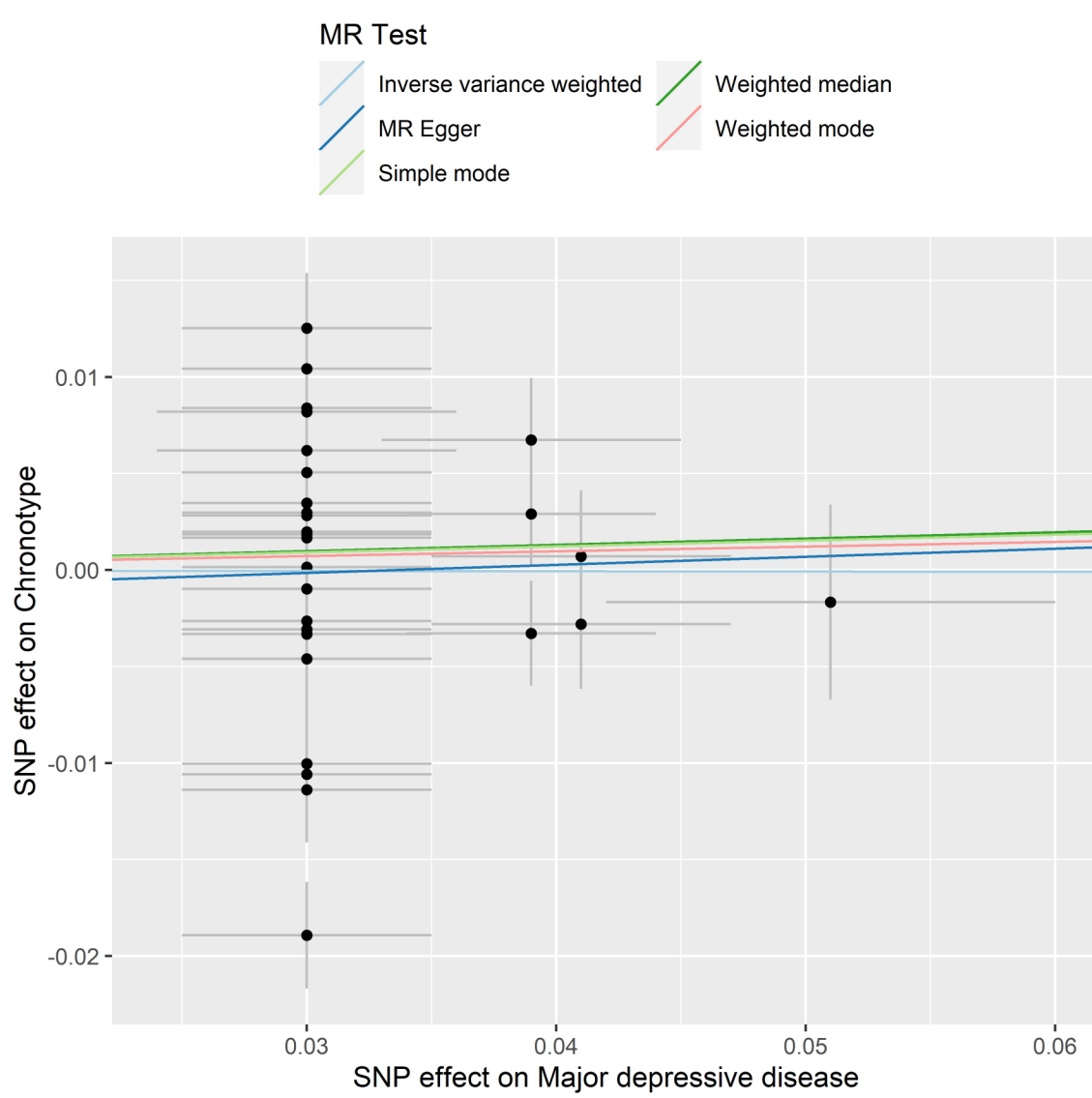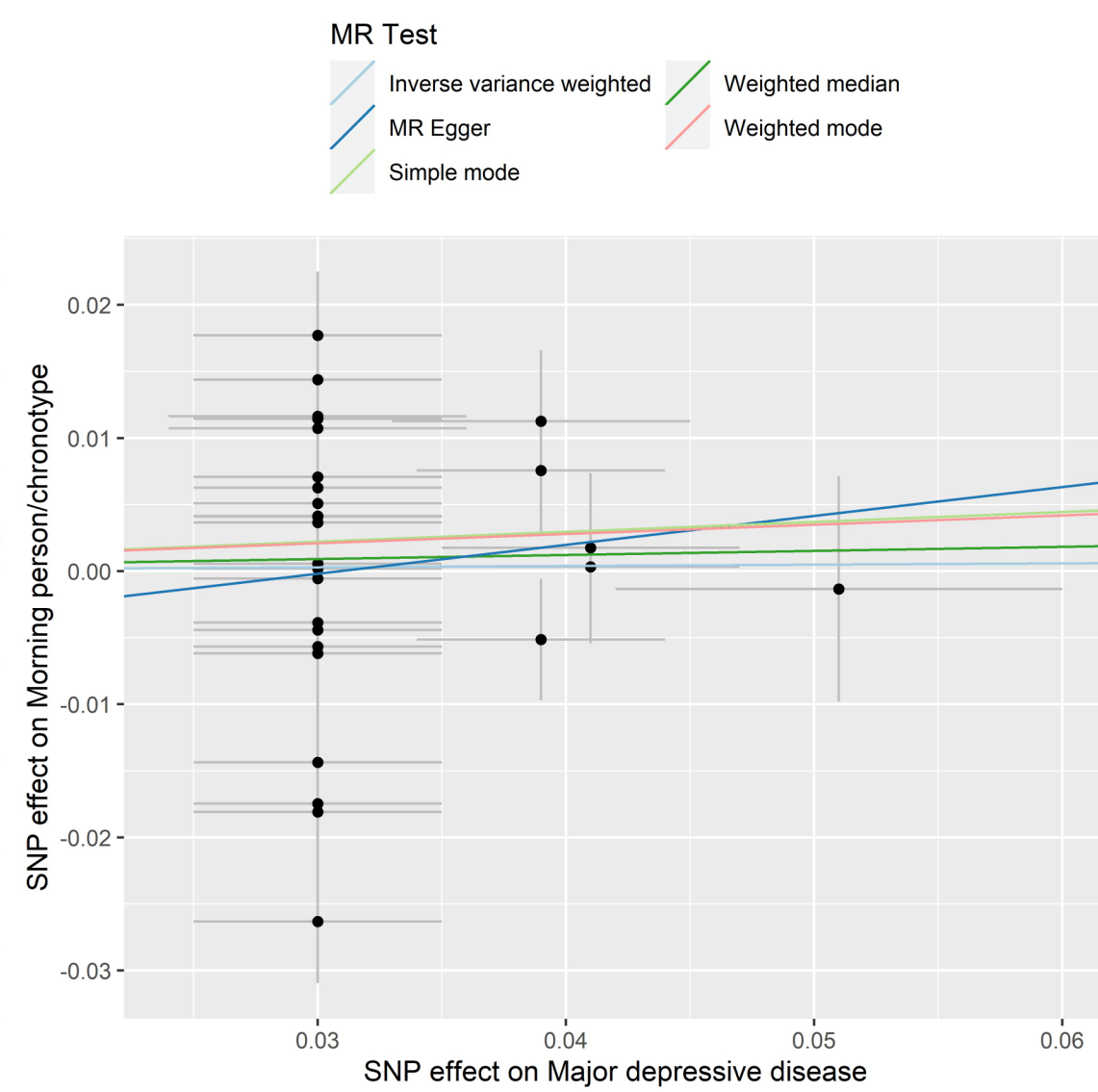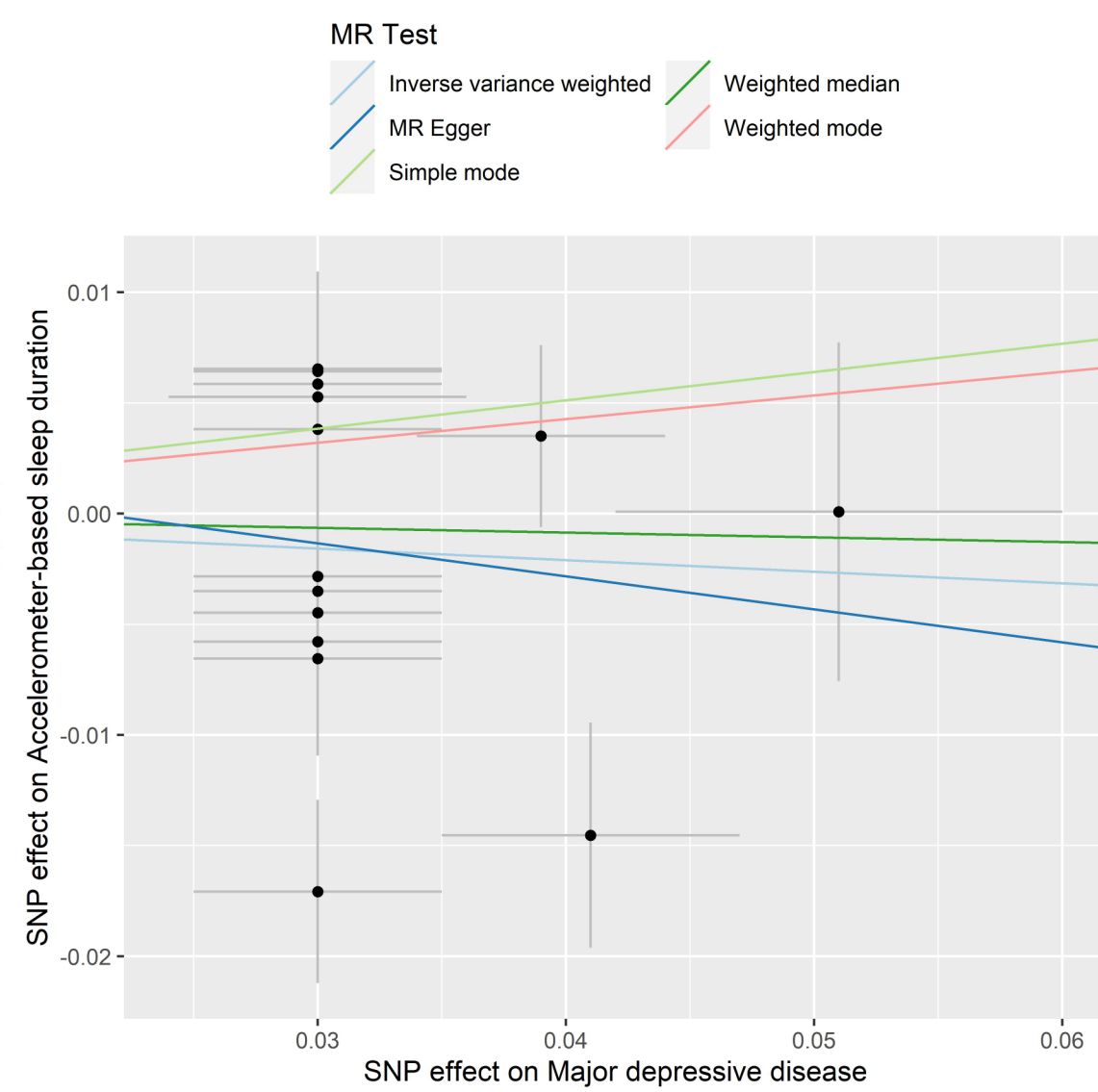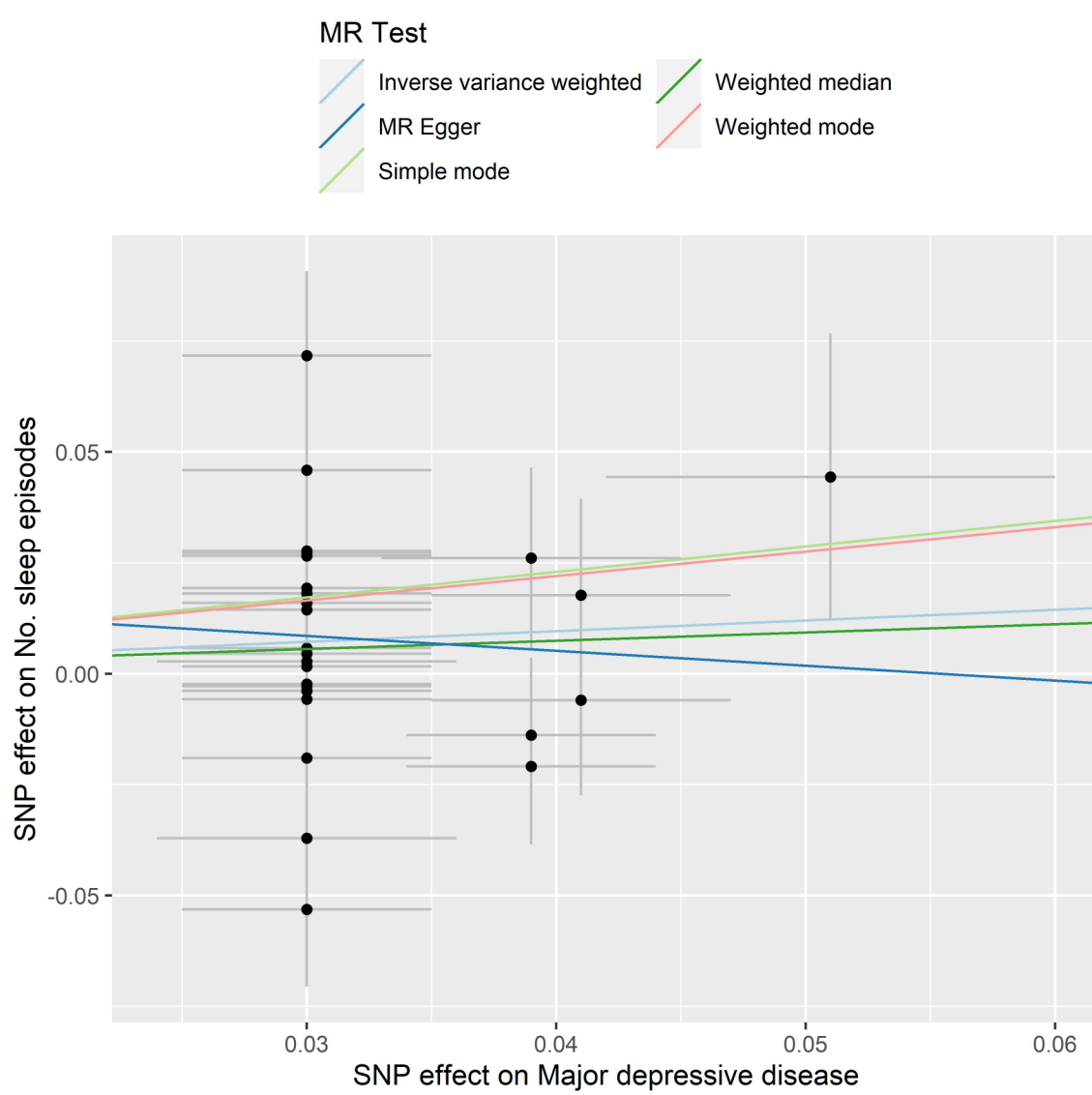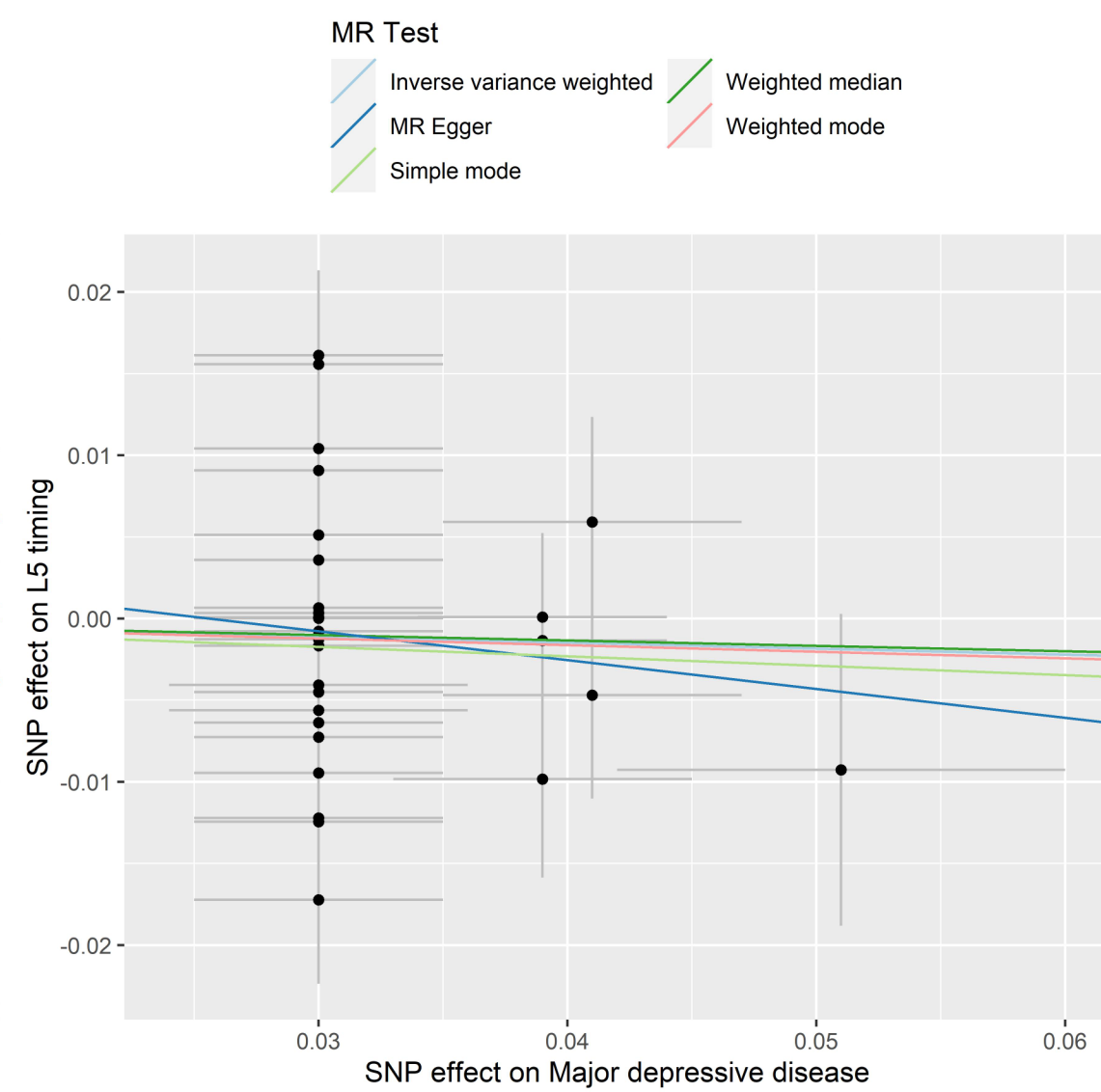

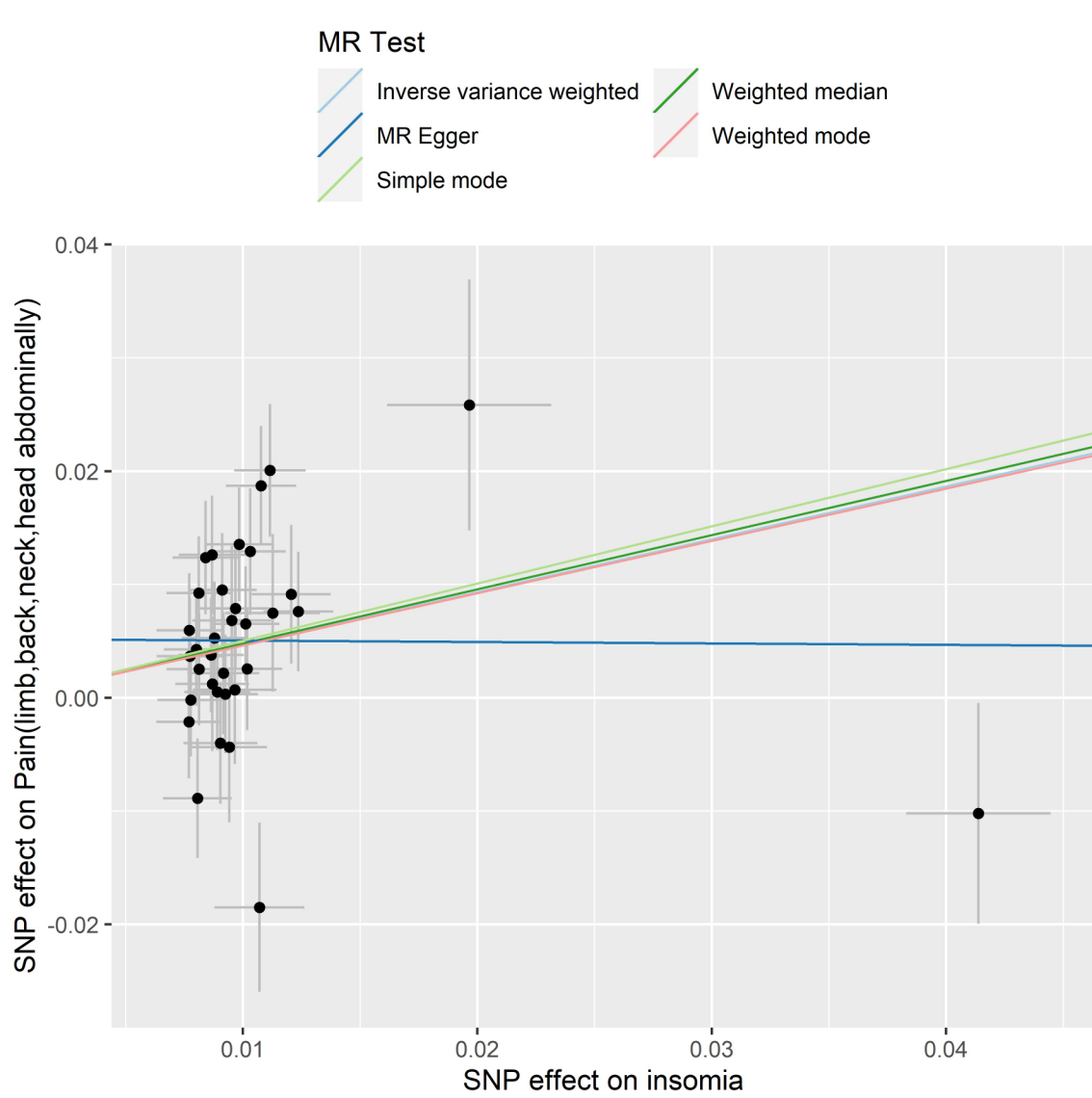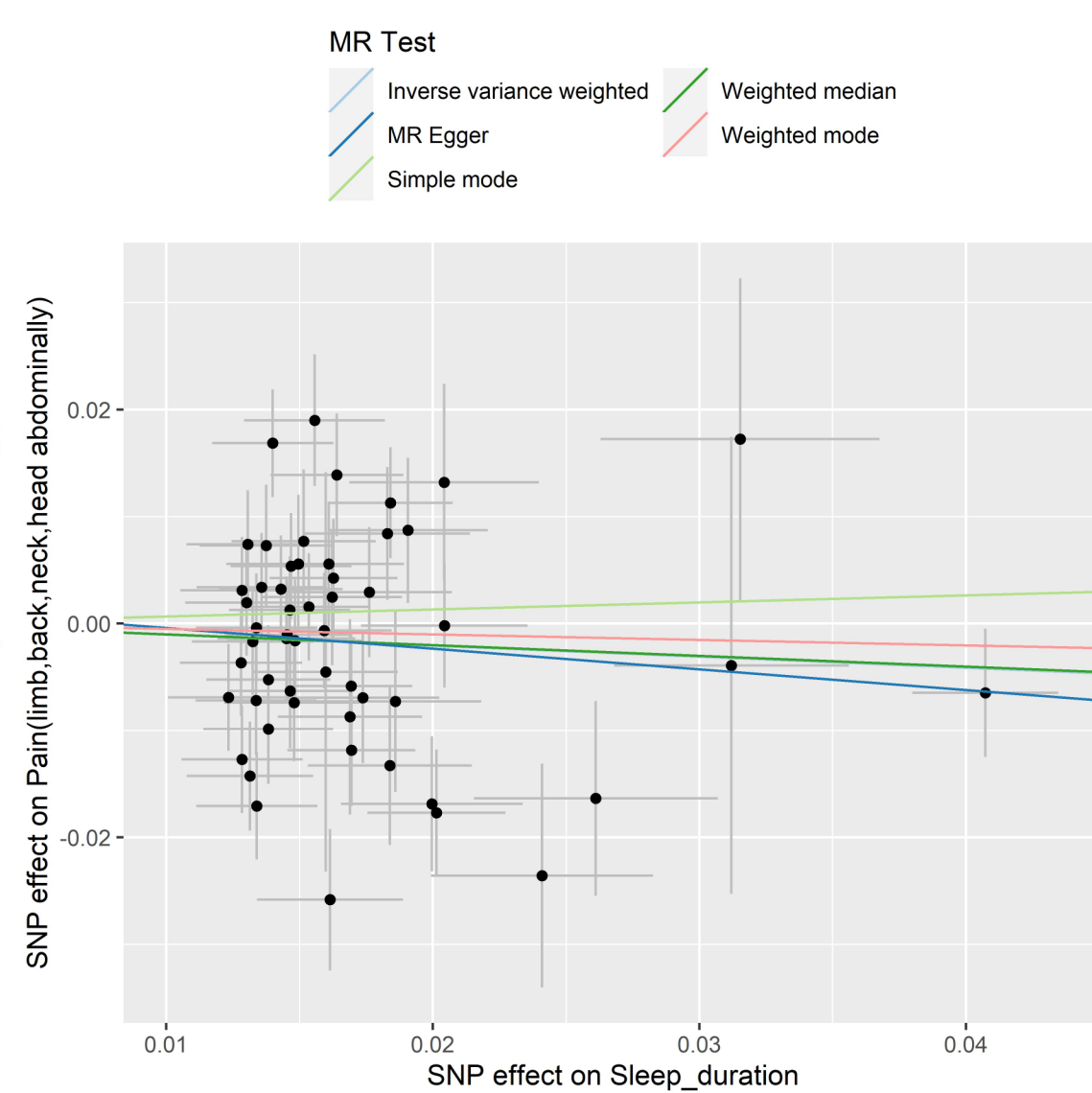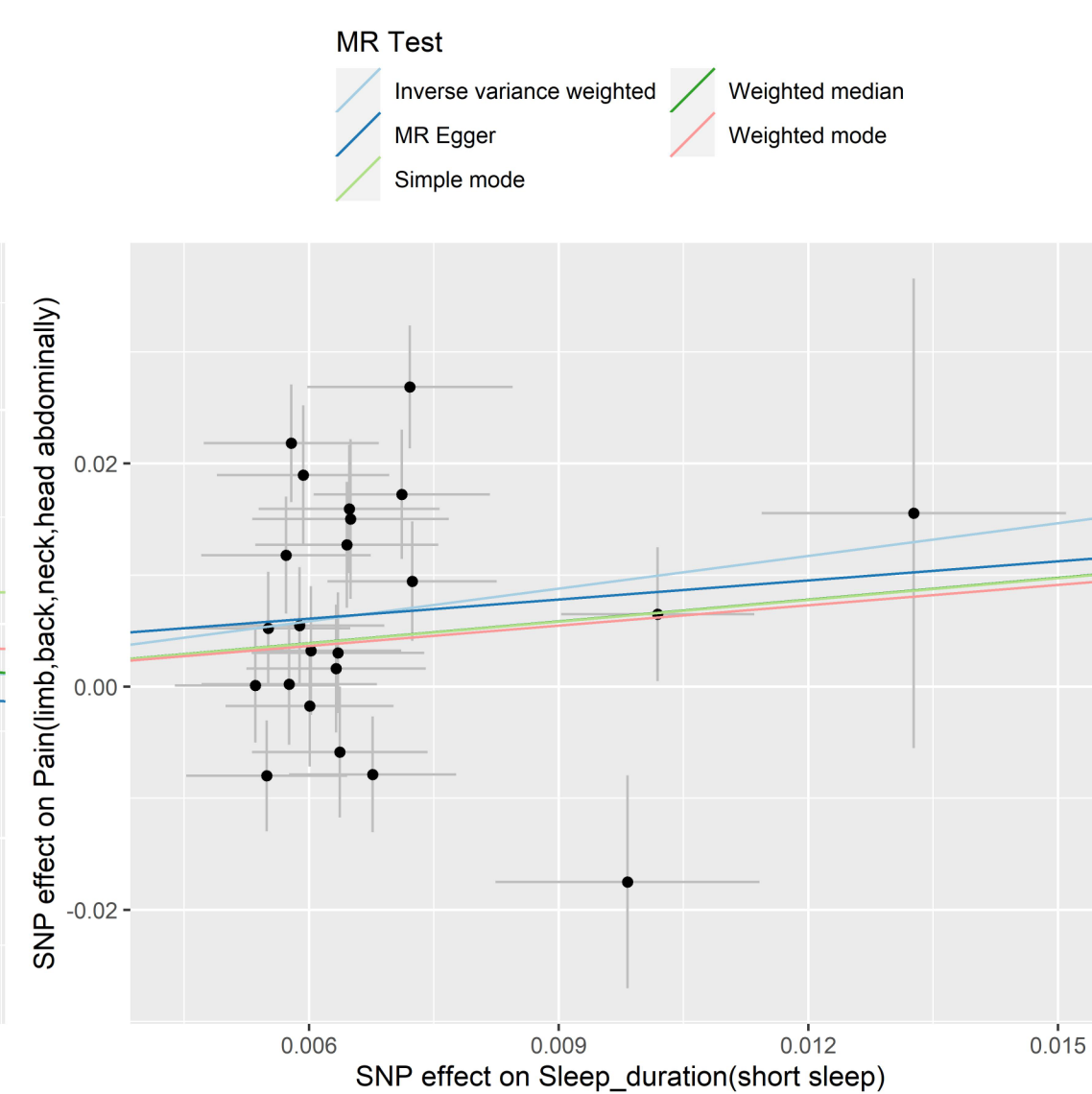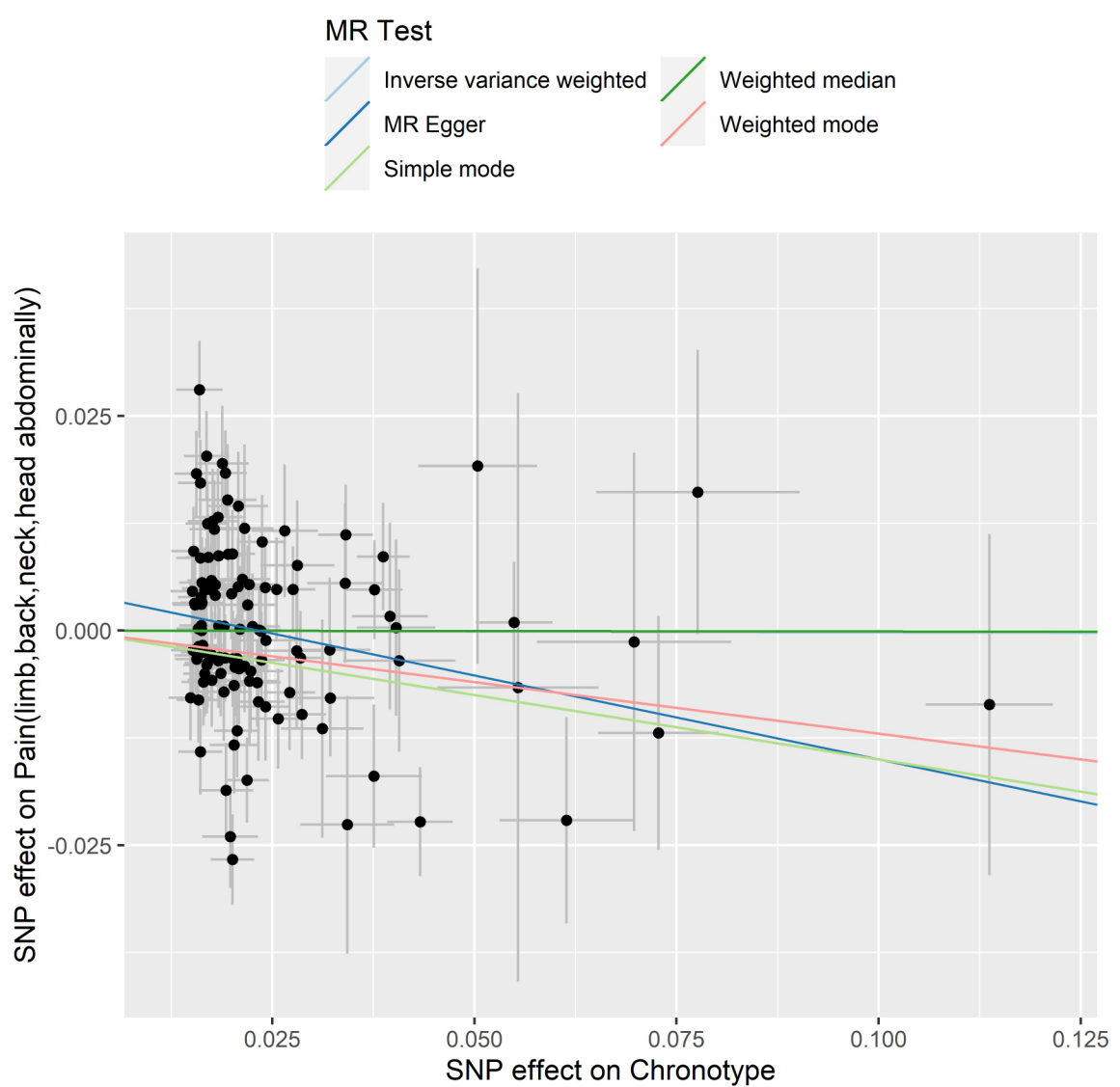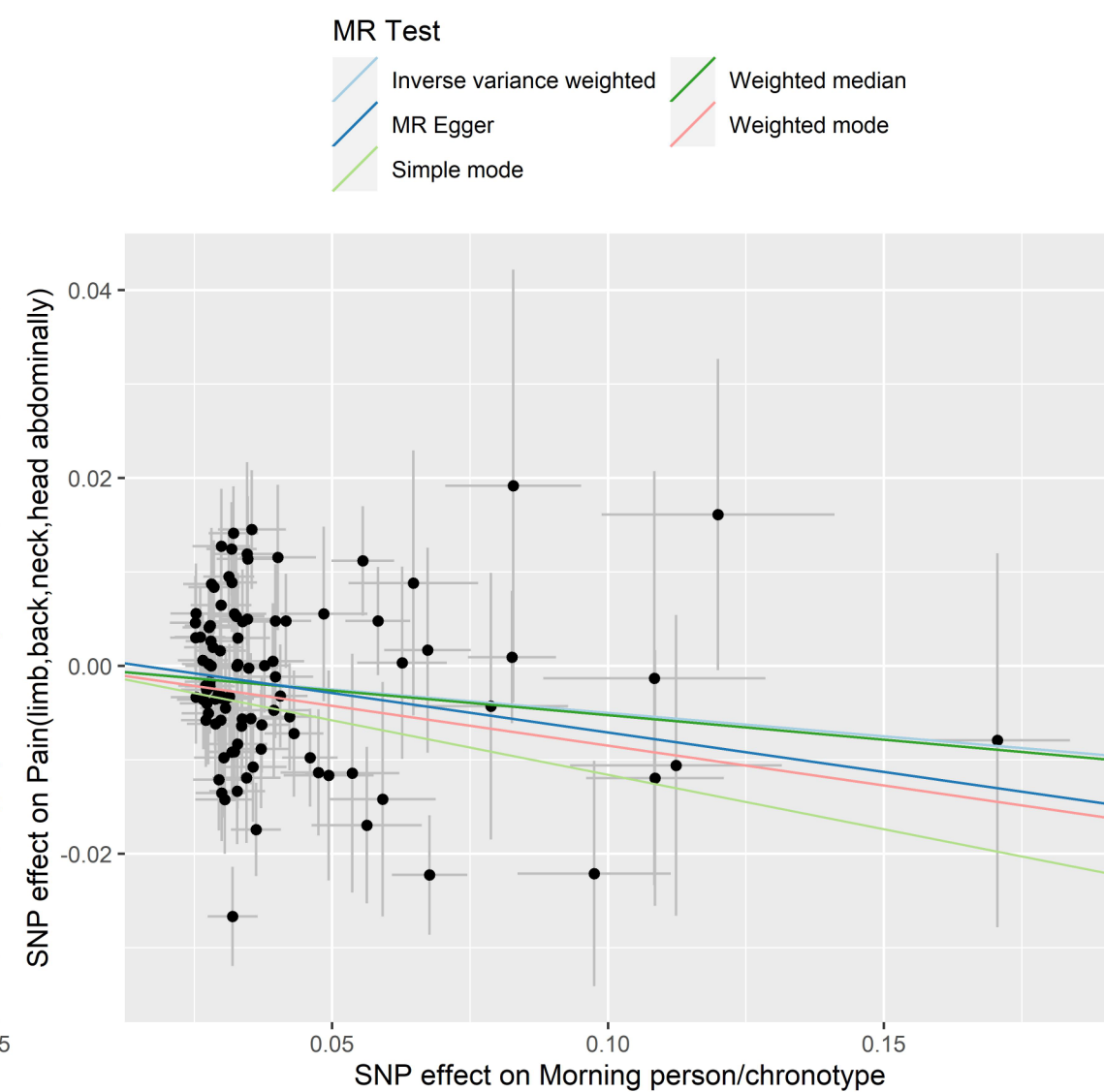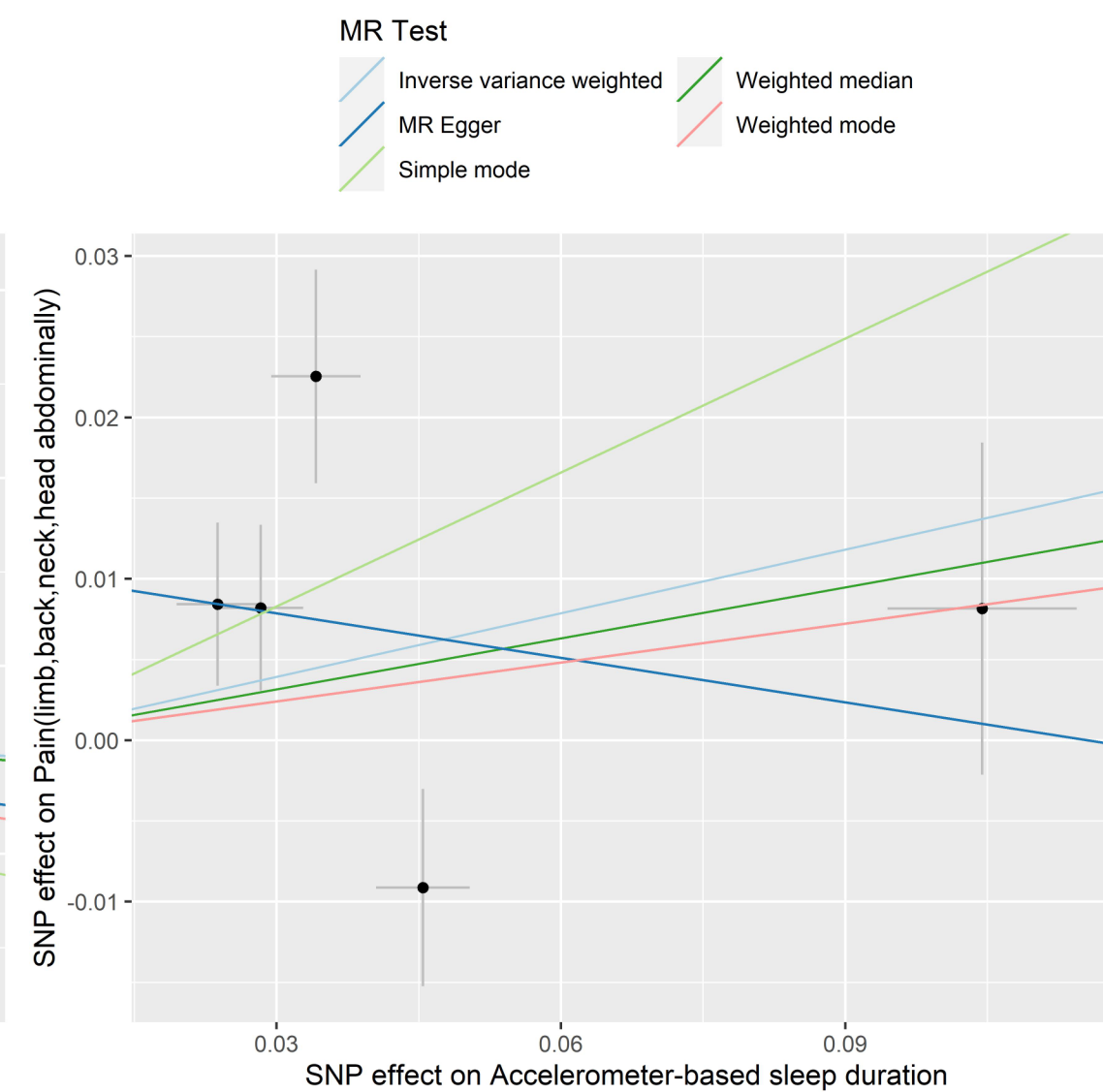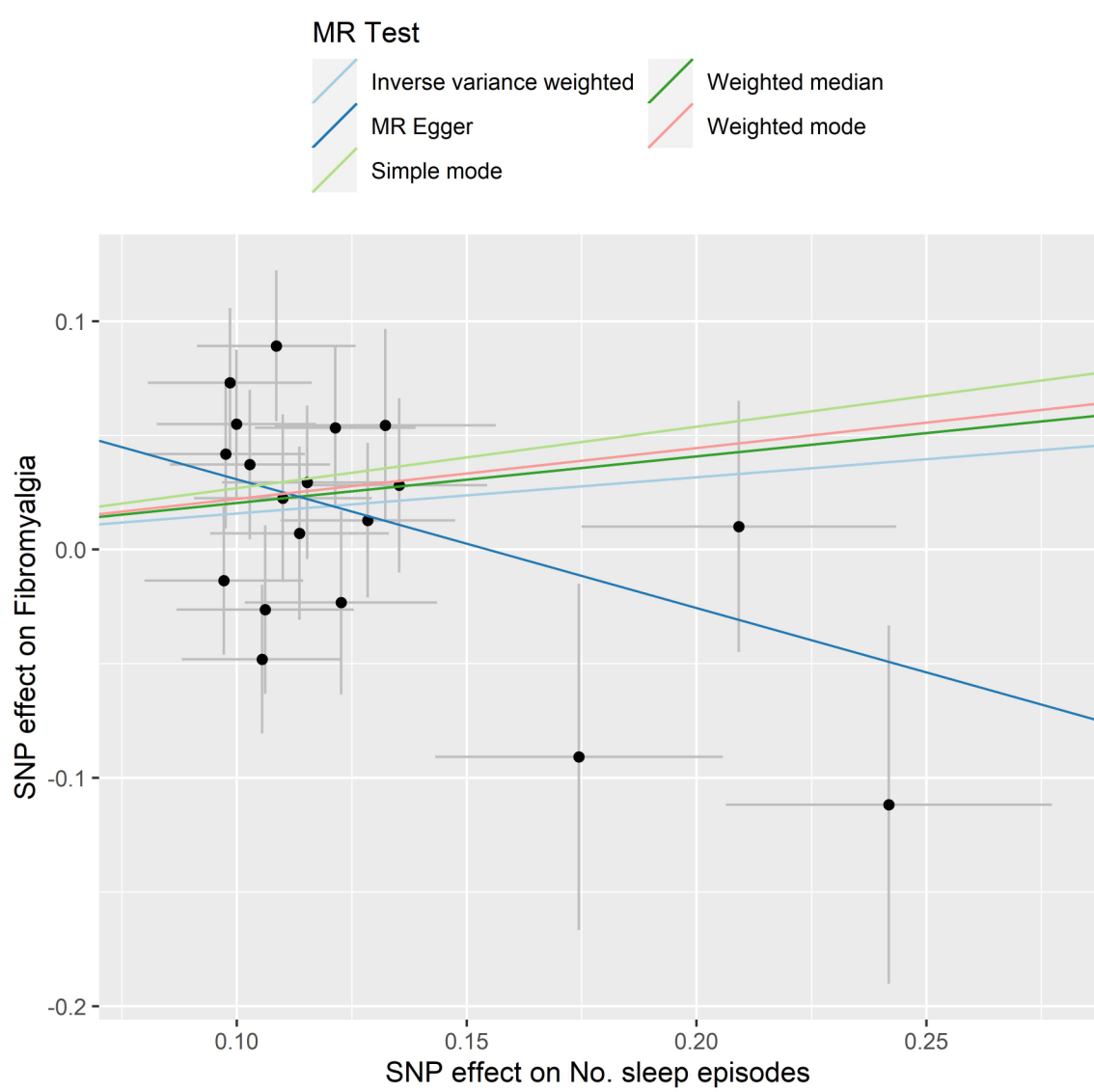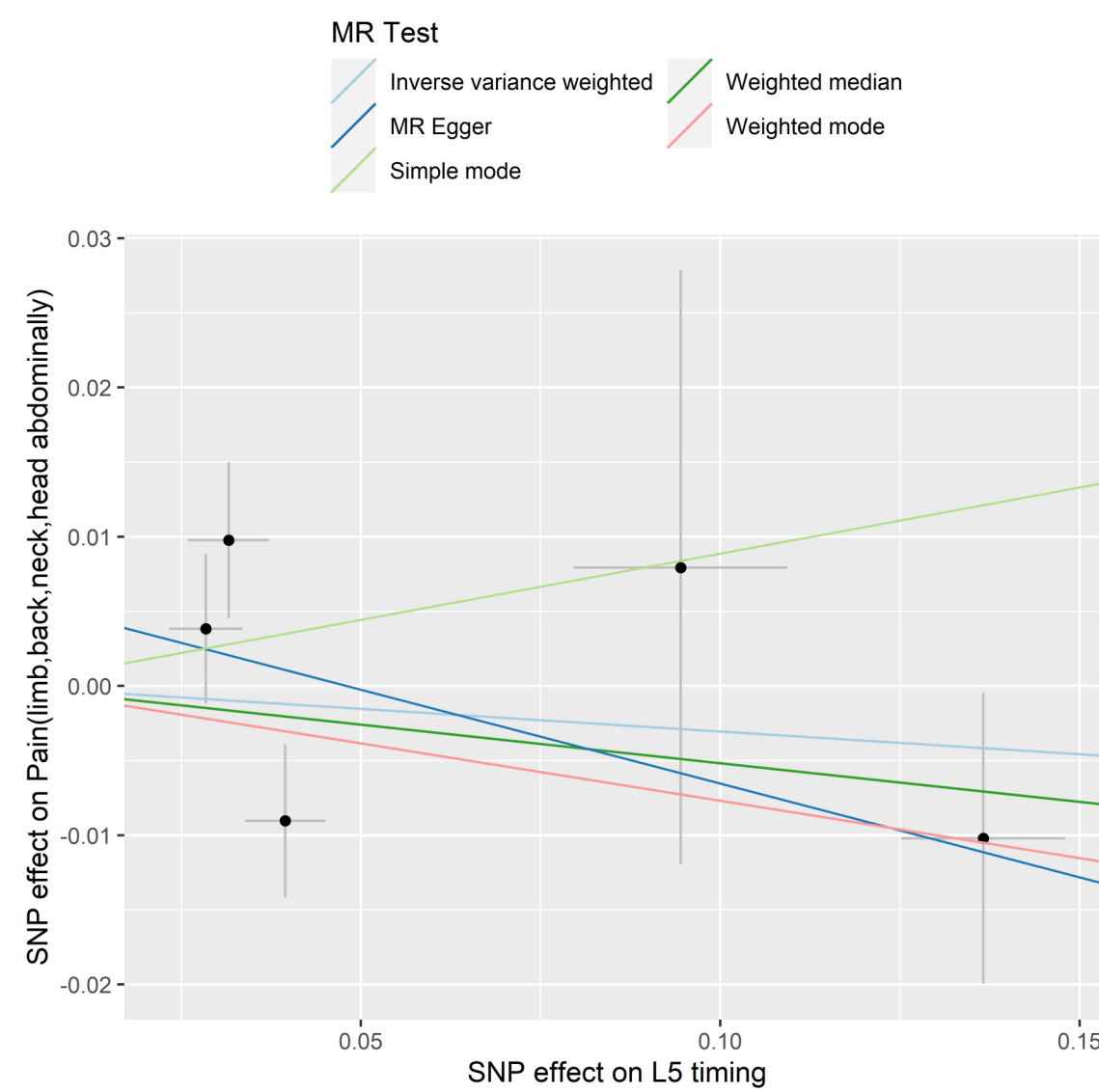

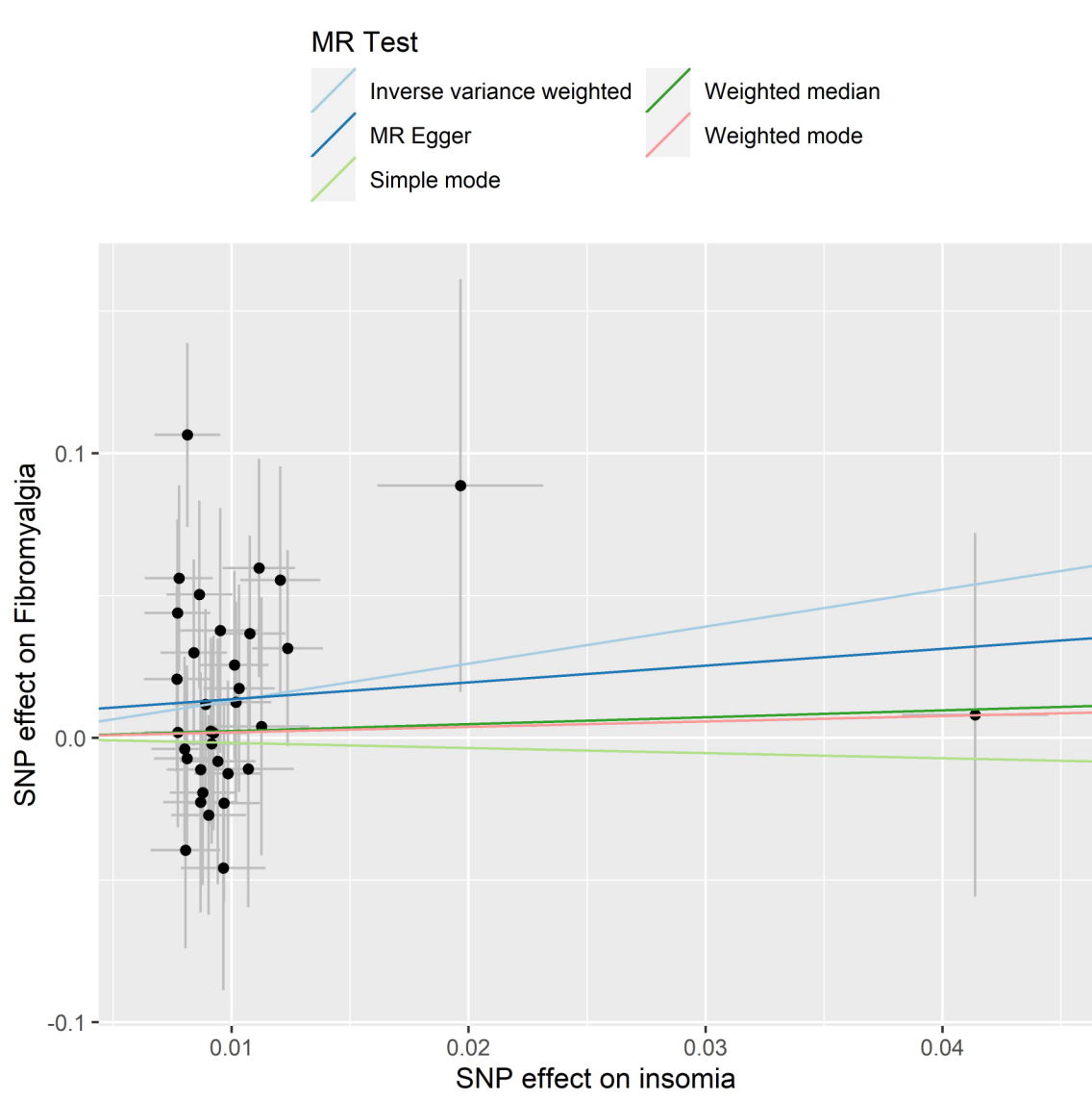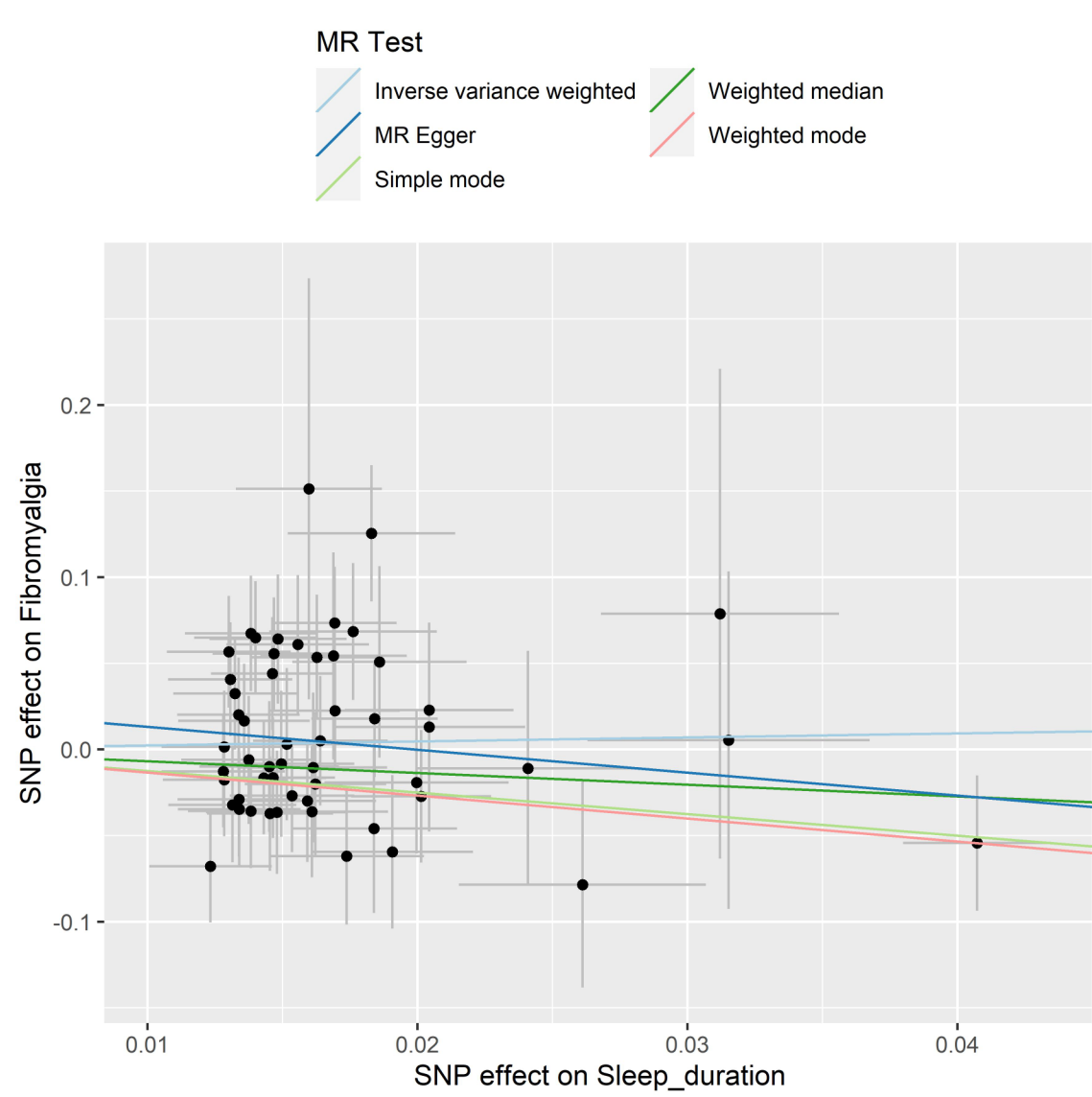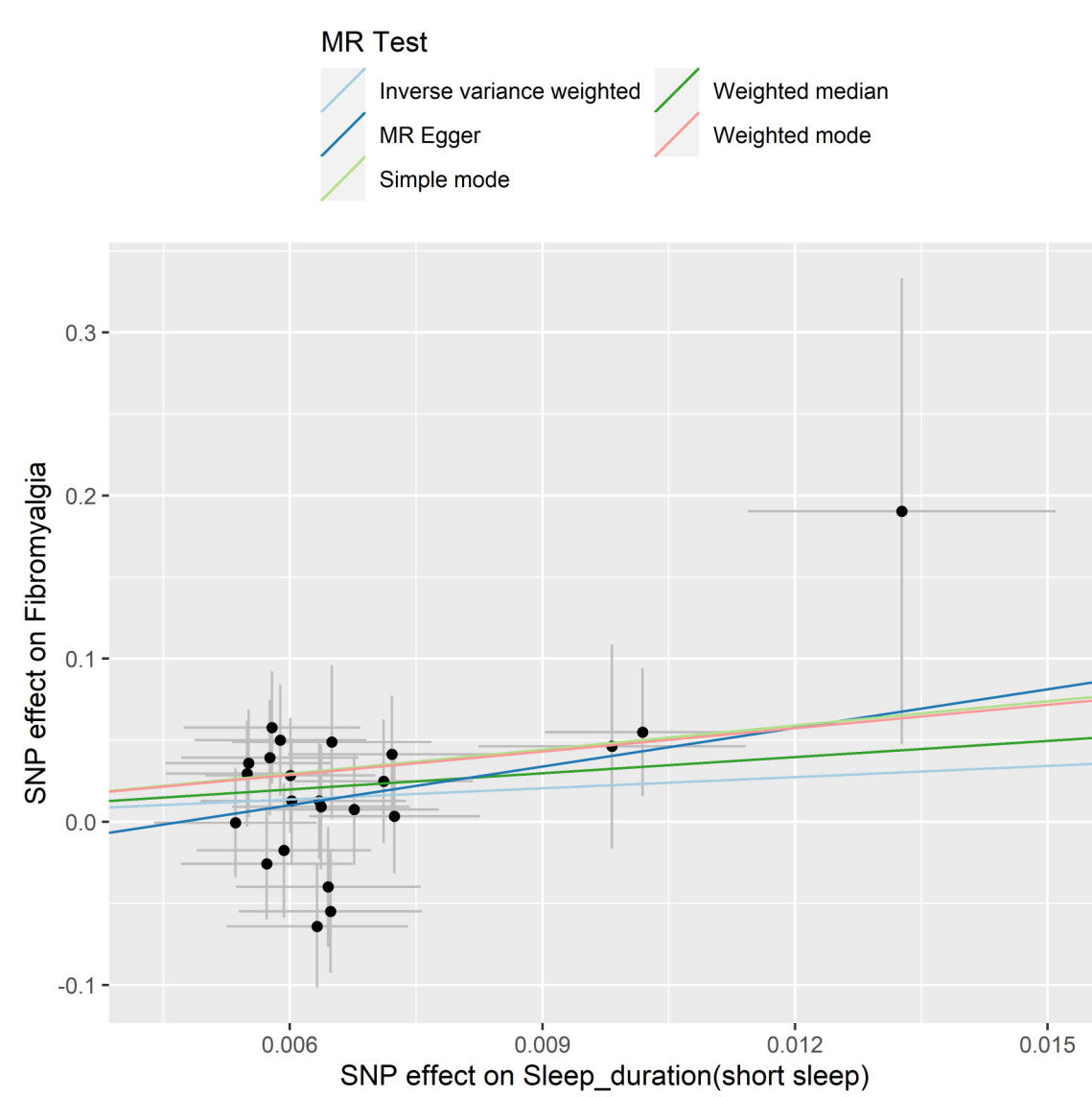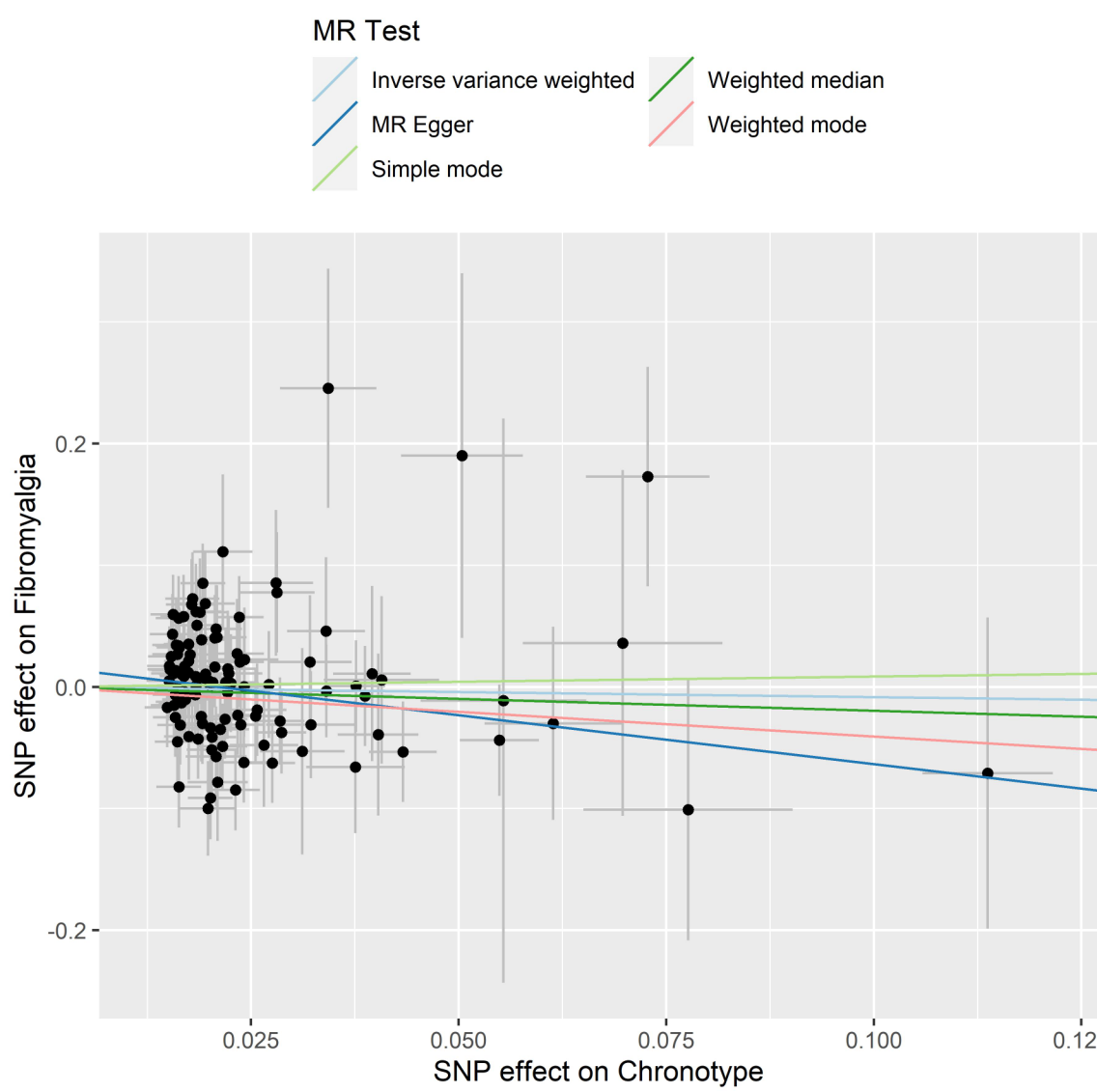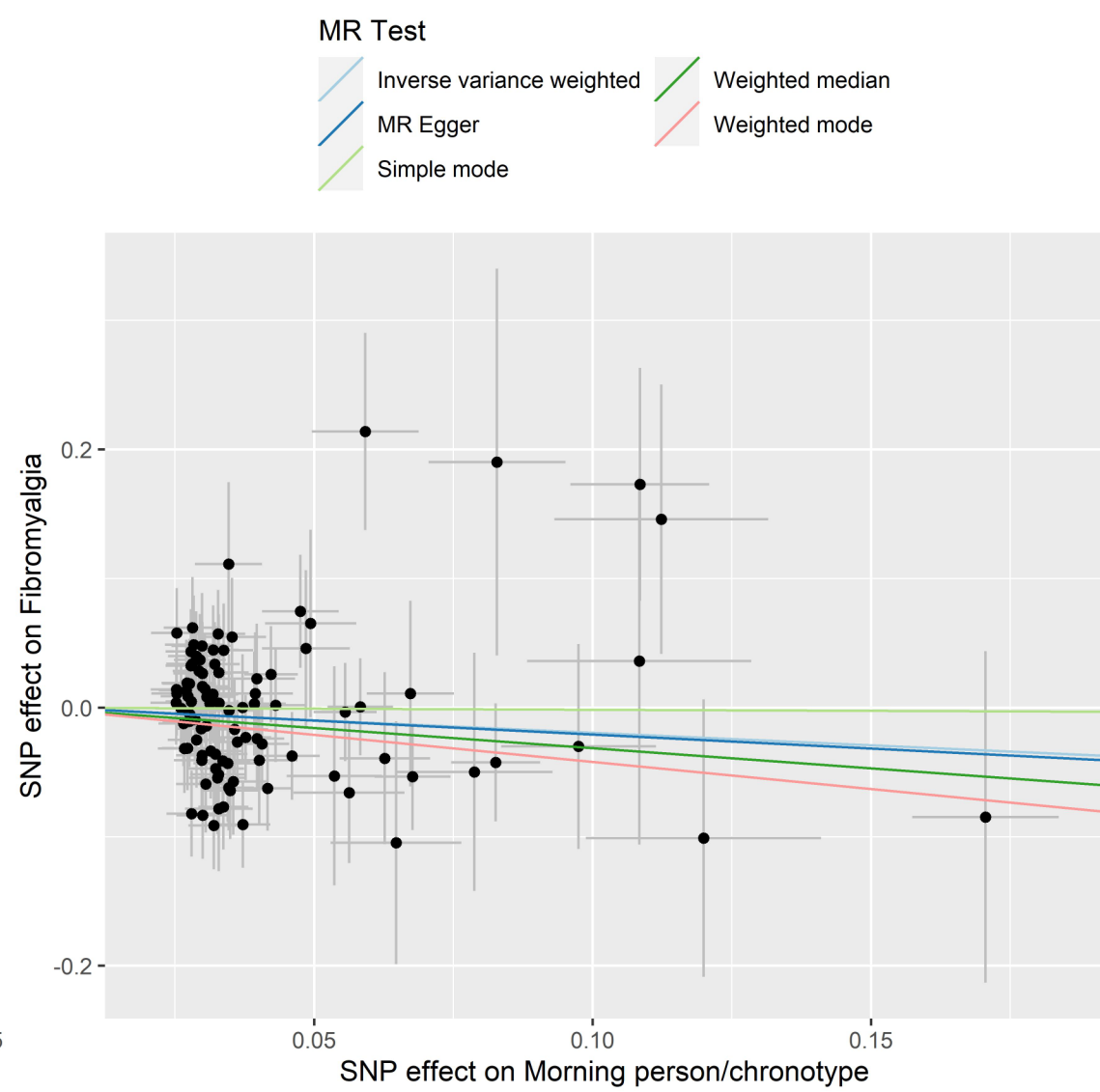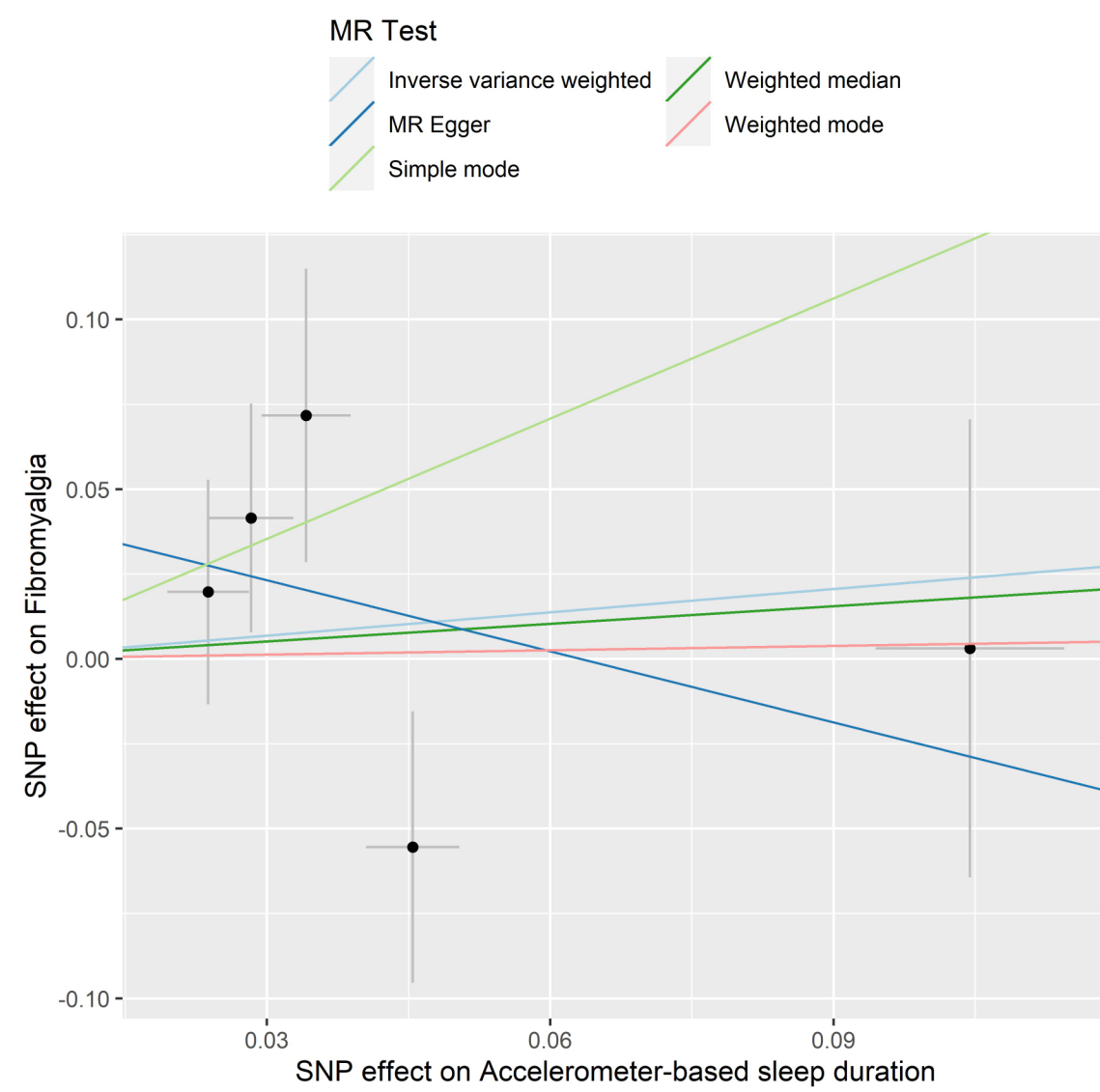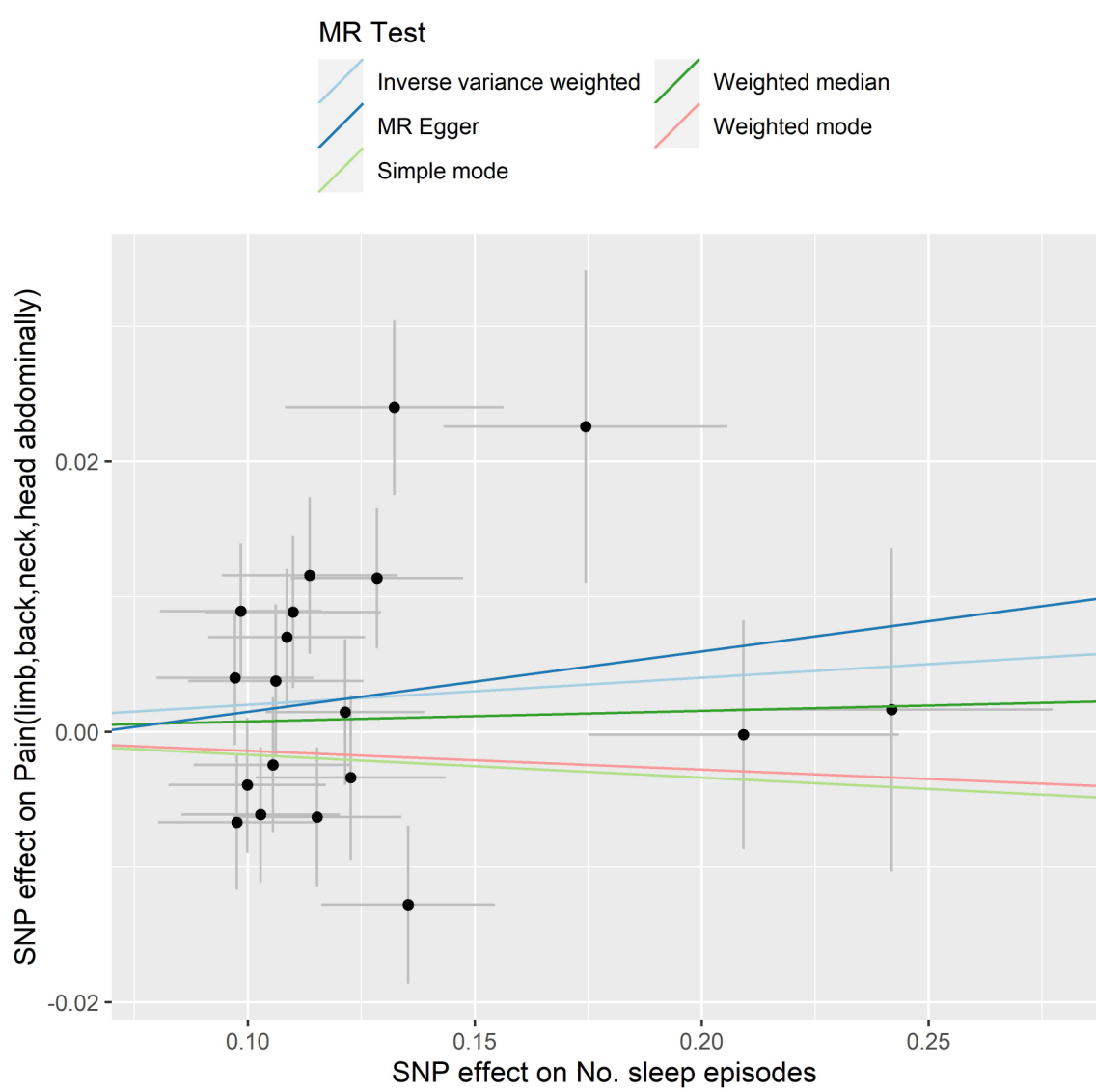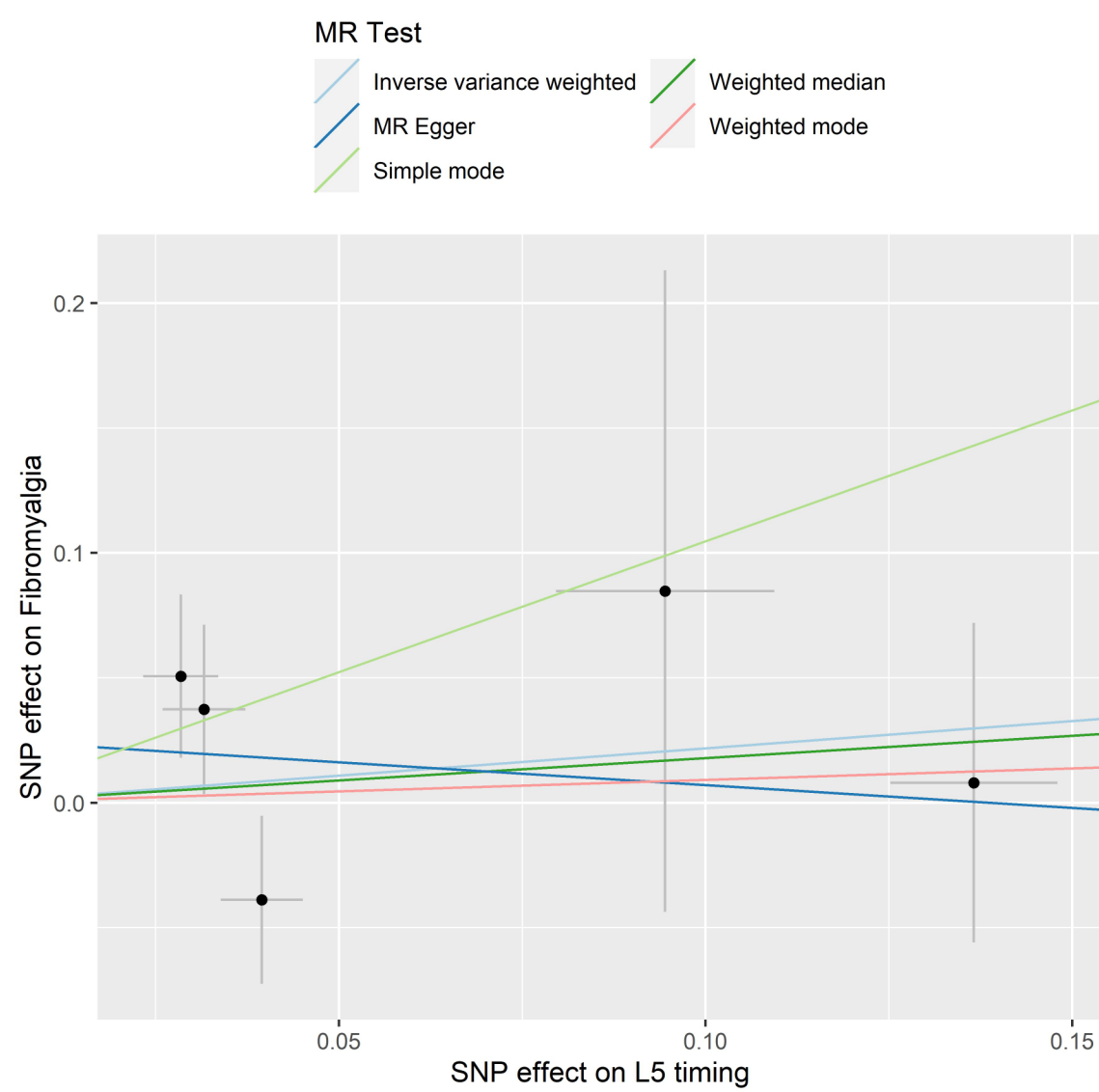

Supplement: Supplementary file 2 — Supporting Information [file BRB3-14-e3596-s001.pdf]
